# From by-products to new application opportunities: the enhancement of the leaves deriving from the fruit plants for new potential healthy products

**DOI:** 10.3389/fnut.2024.1083759

**Published:** 2024-06-04

**Authors:** Lucia Regolo, Francesca Giampieri, Maurizio Battino, Yasmany Armas Diaz, Bruno Mezzetti, Maria Elexpuru-Zabaleta, Cristina Mazas, Kilian Tutusaus, Luca Mazzoni

**Affiliations:** ^1^Dipartimento di Scienze Agrarie, Alimentari ed Ambientali – Università Politecnica delle Marche, Ancona, Italy; ^2^Research Group on Foods, Nutritional Biochemistry and Health, Universidad Europea del Atlántico, Santander, Spain; ^3^Department of Clinical Sciences, Polytechnic University of Marche, Ancona, Italy; ^4^International Joint Research Laboratory of Intelligent Agriculture and Agri-Product Processing, Jiangsu University, Zhenjiang, China; ^5^Universidad Internacional Iberoamericana, Campeche, Mexico; ^6^Research Center for Foods, Nutritional Biochemistry and Health, Universidade Internacional do Cuanza, Cuito, Angola

**Keywords:** circular economy, leaves, bioactive compounds, health, food industry, cosmetics

## Abstract

In the last decades, the world population and demand for any kind of product have grown exponentially. The rhythm of production to satisfy the request of the population has become unsustainable and the concept of the linear economy, introduced after the Industrial Revolution, has been replaced by a new economic approach, the circular economy. In this new economic model, the concept of “the end of life” is substituted by the concept of restoration, providing a new life to many industrial wastes. Leaves are a by-product of several agricultural cultivations. In recent years, the scientific interest regarding leaf biochemical composition grew, recording that plant leaves may be considered an alternative source of bioactive substances. Plant leaves’ main bioactive compounds are similar to those in fruits, i.e., phenolic acids and esters, flavonols, anthocyanins, and procyanidins. Bioactive compounds can positively influence human health; in fact, it is no coincidence that the leaves were used by our ancestors as a natural remedy for various pathological conditions. Therefore, leaves can be exploited to manufacture many products in food (e.g., being incorporated in food formulations as natural antioxidants, or used to create edible coatings or films for food packaging), cosmetic and pharmaceutical industries (e.g., promising ingredients in anti-aging cosmetics such as oils, serums, dermatological creams, bath gels, and other products). This review focuses on the leaves’ main bioactive compounds and their beneficial health effects, indicating their applications until today to enhance them as a harvesting by-product and highlight their possible reuse for new potential healthy products.

## 1 Introduction

The world population is growing exponentially, now reaching 8 billion, and with it, the demand for products of any kind; the consumption of products inevitably leads to the production of waste. The food industry produces a large quantity of waste and therefore, one of the main objectives of the European Union (EU) against food waste and towards sustainable development is waste management and valorization (1, 2). Each year, about 931 million tons of food become waste and the Food and Agriculture Organization of the United Nations (FAO) has estimated that food loss and waste cost the global economy $936 billion a year. 61% of this waste comes from households, 26% from food services, and 13% from retail, according to the UN Environment Programme’s (UNEP) Food Waste Index Report, 2021 (3). The production of waste and by-products greatly impacts the economic, social, and environmental sectors. As for the latter, they are the main factors contributing to the global problems of biodiversity loss, pollution, and climate change. Food waste is a significant contributor to Green House Gas (GHG) emissions (4). Much of this unused biomaterial ends up in city landfills, causing serious environmental problems. From an economic point of view, the negative impact is related to the costs associated with solid waste management and landfills. Moreover, handling large quantities of various degradable substances is a major challenge (5, 6). For all these reasons, efficient waste management is becoming one of the most important challenges to face in the 21st century in the agri-food sector.

The European Commission has defined food waste as one of the priority areas of the Action Plan for the European Circular Economy Strategy (1). The circular economy (CE) is a strategy that may be able to overcome the critical issues described (7). The concept of CE arises from the idea of emulating nature, closing the circuits, and thus, creating complete cycles applicable to a functional and sustainable economic system (8). The CE is based on the classic 3Rs (Reduce, Reuse, and Recycle) and in recent years other 3Rs have been added: Redesign, Rebuild, and Recover (9). The objective of CE is to reduce the introduction of raw materials into the production system, reuse, recycle, or recover the residues produced by the various industries, and then, apply the concept of circularization to create new economically favorable production models (10). By limiting the overuse of raw materials and energy and avoiding the generation of unnecessary waste, CE changed dramatically the “take-make-dispose” paradigm towards a recovery and regeneration system (11, 12). The transition to CE will affect many social, economic, and environmental areas, creating opportunities for revitalization, renewal, and innovation in the agri-food industry and protecting resource scarcity (13). In other words, an important goal of the economic cycle is to develop and close material cycles for better waste prevention and better management of natural resources (14–16). The use and improvement of agricultural by-products such as leaves, grains, seeds, peels, and roots are very important to prevent the overuse of natural resources. These materials can be seen as widely available and inexpensive sources of value-added compounds, not only of energy to produce biofuels but also of added-value compounds, the recovery of which represents a precious opportunity (17).

For the past few years, consumer food waste has only been considered a problem in developed countries, including losses during production, storage, and transportation. However, according to a UNEP report, household food waste *per capita* is similar in high, upper-middle, and lower-middle-income countries. Fruit and vegetable waste (FVW) includes the inedible parts of food sources discarded during collection, handling, transport, and processing (18). In 2014, the European Commission defined the term “food waste” as “food (including inedible parts) lost from the food supply chain, not including food diverted to material uses such as bio-based products, animal feed, or sent for re-distribution” (19). FVW production occurs at all stages of the food supply chain, but the amount produced at each stage varies greatly from country to country, depending on the level of development in the area being studied. Collection and processing are the most important steps in the production of FVW in developing countries (20). Process by-products are also found in food waste if they are not used for other essential functions (21), but they are often recycled by industry. The Waste Framework Directive (1) emphasizes the importance of preventing the generation and exploitation of waste through reuse/recycling, with industrial by-products playing an important role in the recycling phenomenon. By-products are defined as secondary products resulting from the production of the main product, often having a market value (22). The scientific community and recognized global organizations such as FAO largely agree that the agricultural industry is one of the largest producers of by-products (23). This industry produces them in the form of leaves, peels, grains, pomace, unripe, and/or damaged fruits and vegetables. As consumers become more informed and conscious, they are calling for the production of “natural” and “organic” products with “green” labels that are considered safe and healthy (24). With this in mind, knowing that fruit and vegetable by-products (FVB) are a source of several high-value-added compounds, these by-products can be used as natural food ingredients or as starting material for innovative food products (25, 26).

### 1.1 Food waste and by-products as a source of bioactive compounds

In general, most fruits and vegetables are only consumed for their pulp, but many studies have found significant amounts of phytochemicals in the seeds, leaves, skins, and other compounds of fruits and vegetables not normally eaten, as well as essential nutrients (27, 28). FVW contains dietary fiber, flavors, fragrances, enzymes, organic acids, and proteins in addition to phenolic compounds. Based on the spectrum of their biological activity, natural products are considered attractive. Among the bioactive compounds that may be present in various agricultural by-products, phenolic compounds may have a beneficial effect on human health, for example, in the prevention of cancer, cardiovascular and neurodegenerative diseases (29–35). These beneficial effects are due in part to their ability to act as potent antioxidants as reactive oxygen species (ROS) scavengers, which are produced under conditions of oxidative stress and numerous inflammatory and degenerative diseases (36–39). These properties suggest the use of natural phenolic compounds in the food industry (40–45) and as food additives (34, 46–49), but also as biomedical (50–52), and cosmetic (53–56) additives. The beneficial properties of these bioactive compounds are described in the scientific literature. Several reviews have summarized the anticancer, antidiabetic, antihypertensive, anti-inflammatory, antibacterial, antioxidant, immunomodulatory, or neuroprotective properties of plant secondary metabolites (SMs) extracted from agricultural products, emphasizing their importance in the human diet (57–61). The antioxidant and antimicrobial properties of seeds, leaves, flesh, peel, and pulp waste have been investigated to develop new functional foods that improve human health and well-being (62–66). Compounds extracted from fruit waste (FW) are finding further use as food additives to maintain and improve quality, prevent food oxidation, and inhibit the growth of pathogens (67). In addition, FW has been used as a partial replacement for wheat flour (68), added to cakes (69), and in beverage production (70). Interest in natural products has also grown in the cosmetics and pharmaceutical industries, where they are a valuable solution for valorizing disposable by-products. The search for new drugs and increased resistance to existing ones has led to the discovery of new sources of antimicrobials, such as bioactive compounds derived from fruit waste (71, 72).

**Figure 1:**
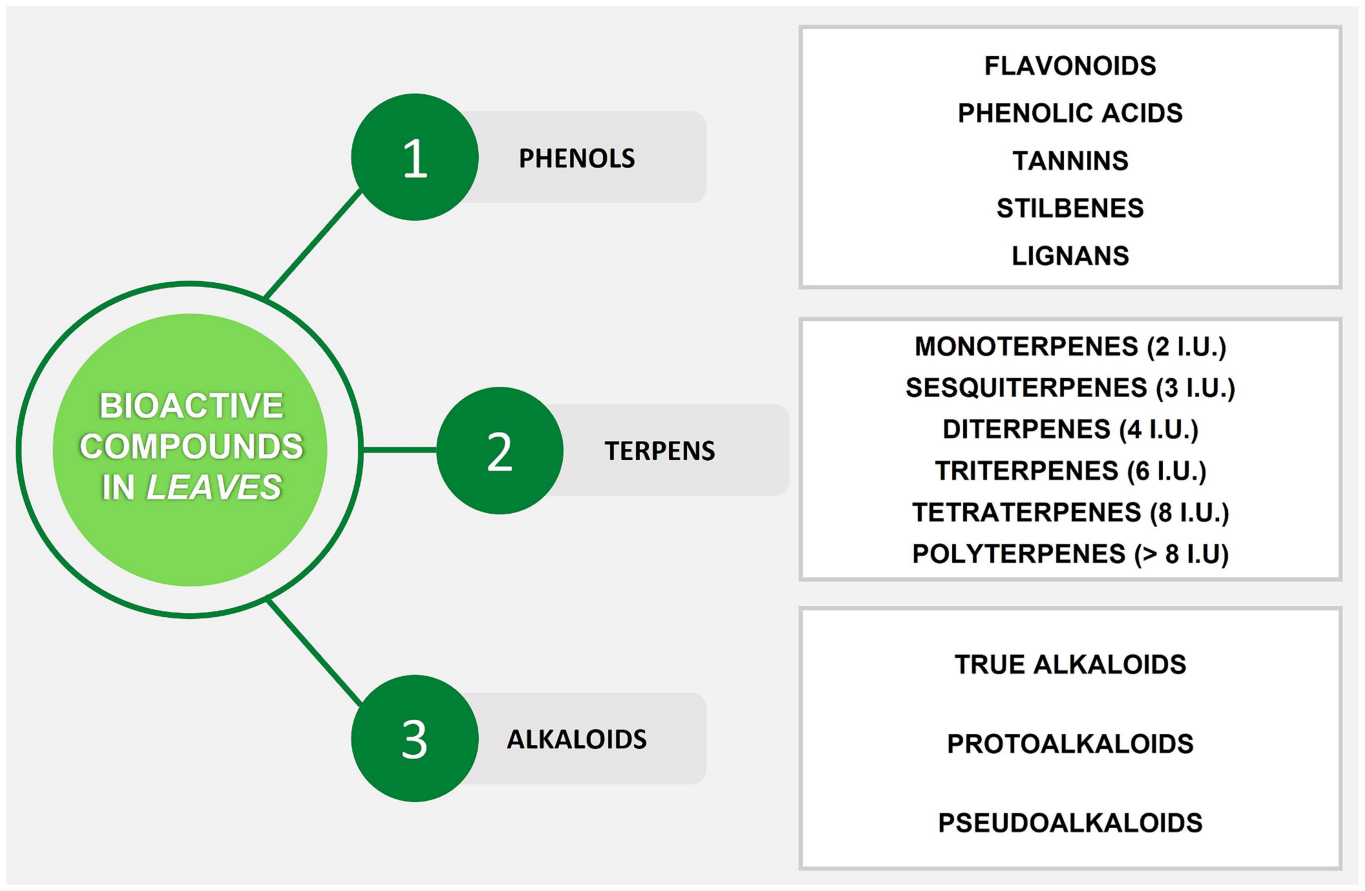
Summary scheme of bioactive compounds in plant leaves. I.U., Isoprene Units.

Secondary metabolites (SMs) are usually divided into three main groups according to their biosynthetic pathways: phenols, terpenes, and alkaloids (73) (Figure 1). The most common pathways responsible for the biosynthesis of SMs are performed through shikimic acid for phenols, aromatic alkaloids, and tannins, acetate-malonate for alkaloids and phenols, and mevalonic acid for steroids, alkaloids, and terpenes (74). These phytochemicals are characterized by great chemical and biological diversity, and their production depends on biotic and abiotic factors. Biotic factors are microorganisms like viruses, bacteria, fungi, and larger animals like herbivores and pollinators; meanwhile, abiotic factors are identified with the characteristics of the environment including temperature, salinity, water, radiation, chemical stress (like mineral salts), and mechanical stress (like wind) (75, 76). In more detail, phenolic compounds are considered one of the main classes of bioactive compounds involved in the organoleptic properties of plants and the nutritional value of fruits and vegetables. They contain one or more aromatic rings along with one or more hydroxyl groups in their basic structure. Polyphenolic compounds are divided into different classes such as flavonoids (subclasses: flavones, flavonols, isoflavones, flavanones, anthocyanidins, flavanols), stilbenes, tannins, phenolic acids, and lignans. Phenolic acids may be present in edible plants as esters or glycosides in combination with other natural compounds such as alcohols, flavonoids, sterols, glucosides, and hydroxy fatty acids. Indeed, the leaves of fruit trees contain large amounts of these bioactive compounds (28). The second group of SMs is terpenes, also called terpenoids or isoprenoids, which constitute a large family of natural products that are very diverse in function, structure, and properties. These compounds are considered to be the basic building blocks of terpenes, as they decompose to form isoprene units (C5). For this reason, these compounds are classified according to the number of C5 units in their structure. The terpenoids found in all plants include pigments (chlorophylls, carotenoids), electron carriers (plastoquinone, ubiquinone), membrane components (sterols), and hormones (gibberellins, abscisic acid, steroids, strigolactones) (77). Along with chlorophylls, carotenoids are essential pigments in the leaves, and they belong to tetraterpenes which consist of eight isoprene units with a 40-carbon skeleton (78). Terpenes may have a wide range of functions in plants, such as attracting pollinators and protecting damaged tissues from attack by insects, parasites, and herbivores. A large number (over 8,000 molecules) of plant SMs are classified as alkaloids (79). The presence of nitrogen in the structure is a chemical property of these compounds. Compared to most other classes of SMs, alkaloids are characterized by great structural diversity, which makes difficult their arrangement into a uniform classification. Alkaloids are generally classified according to the starting substances of their biosynthetic pathways, such as the amino acids that provide nitrogen atoms and part of their skeleton including purines and terpenoids. According to this classification, there are three main types of alkaloids: (i) true alkaloids, (ii) protoalkaloids (both deriving from amino acids), and (iii) pseudoalkaloids (not deriving from amino acids) (80). These SMs are a huge group of environmentally important phytochemicals with multiple pharmacological, toxicological, cosmetic, and nutritional activities. However, the presence and distribution of these metabolites depend on the stage of the plant life cycle and vary greatly among plant species, producing different types of alkaloids that accumulate in different organs such as leaves. Alkaloids play many roles in plants as they are involved in protection and pollinator attraction. Many alkaloids are toxic to various organisms, protect plants from pathogens, and prevent grazing by non-specialist herbivores (76).

### 1.2 Antioxidant, antibacterial, and anticancer effects of leaves

Traditionally, plant-based products have been used for different purposes. Several centuries ago, people around the world employed different parts of plants, including leaves, as a natural remedy for various pathological conditions (81). Numerous reports regarding the use of leaf products for the treatment of many human diseases have been made (82–84). Recently, a lot of research has been conducted on leaves of numerous plant species about antioxidant and other biological activities and their potential use in the food industry as potent antioxidants and as food, biomedical, and cosmetic additives. Proven medicinal properties include mainly antioxidant, anti-inflammatory, anti-allergic, antibacterial, and antiviral effects.

The process of oxidation is defined as the transfer of electrons from one atom to another. When the flow of electrons is interrupted (unpaired single electron transfer), free radicals are formed. Free radicals known as reactive oxygen species (ROS) include hydroxyl (HO), alkoxyl (RO˙), superoxide (O2^.-^), nitric oxide (NO˙), and peroxyl (ROO˙) (85). Physiological concentrations of ROS may be required for normal cell function. If this balance is destabilized, ROS can also damage important biomolecules such as lipids, nucleic acids, carbohydrates, polyunsaturated fatty acids, and proteins. Oxidative damage to biomolecules can lead to cancer, aging, and many other diseases (86). In recent years, there has been great interest in identifying alternative safe and natural sources of dietary antioxidants, and in the search for natural antioxidants, especially plant-based ones (87, 88). Foods, especially fruits and vegetables, also play an important role in maintaining the physiological redox balance. These foods provide the body with several antioxidants, including vitamin C and some polyphenolic compounds (e.g., resveratrol and flavonoids) (89). Fruit and vegetable by-products, such as leaves, can also be used to obtain compounds useful for maintaining the physiological balance between antioxidant and pro-oxidant substances.

Another beneficial effect of the presence of bioactive compounds in fruit leaves is antimicrobial activity. This is a generic term for all active ingredients/agents that inhibit bacterial growth, prevent microbial colonization, and may kill microorganisms (90). In the literature, there are a lot of studies that demonstrate and confirm the antimicrobial activity of leaves from fruit plants due to the presence of bioactive compounds, mainly phenol derivatives. The most affected strains resulted be *S. aureus*, *S. epidermidis*, *M. luteus*, *E. coli*, *B. subtilis*, *B. cereus*, *P. aeruginosa*, *K. pneumoniae*, *S. marcescens*, *A. tumefaciens*, *S. lutea*, *L. monocytogenes*, and *S. sonnei*, but their susceptibility/resistance varies according to the plant species, the type of extract, and the specific composition of phenolic compounds of leaves (91–95).

Despite it is still a field insufficiently explored by researchers, several studies report potential anticancer effects of leaves from fruit trees on different cell lines, e.g., berry plant leaves extracts against promyelocytic HL60 cell line (96), *Carica papaya* leaves extract on cervical carcinoma (Hela), breast adenocarcinoma (MCF-7), hepatocellular carcinoma (HepG2), lung adenocarcinoma (PC14), pancreatic epithelioid carcinoma (Panc-1), and mesothelioma (H2452) hematopoietic cell lines (97, 98), grape leaves extracts on HepG2 and MCF-7 cancer cells (99), and lingonberry leaves extracts against human clear cell renal cell carcinoma (CaKi-1), human colon adenocarcinoma (HT-29), and human malignant melanoma (IGR39) cell lines (100). Bioactive compounds such as vitamins, pro-anthocyanidins, anthocyanidins, flavonols, and phenolic acids found in leaf extracts may exert antitumor effects.

In this context, our review focuses on the main applications of the leaves until today to use the discarded leaves from fruit plants as a harvesting by-product and highlight their beneficial effects in traditional medicine, in the prevention and treatment of many chronic diseases, and their potential use in chemistry, pharmacology, medicine, and agronomy industries, serving as a link among researchers from different scientific fields. Utilizing electronic databases, such as Medline, Scopus, Google Scholar, and Web of Science, a thorough search was conducted. The list of search terms used for the manuscript is included in the supplementary material. As a result of the literature search, hundreds of research were discovered. The references were chosen to be representative of as many fruit plant leaves as feasible and to be relevant in the literature, published in English between 1980 and 2023. The fruit species selected were subjected to a minimum number of studies which gave us the possibility to dedicate them a chapter.

## 2 Structure, functions, and biochemical composition of leaves

**Figure 2:**
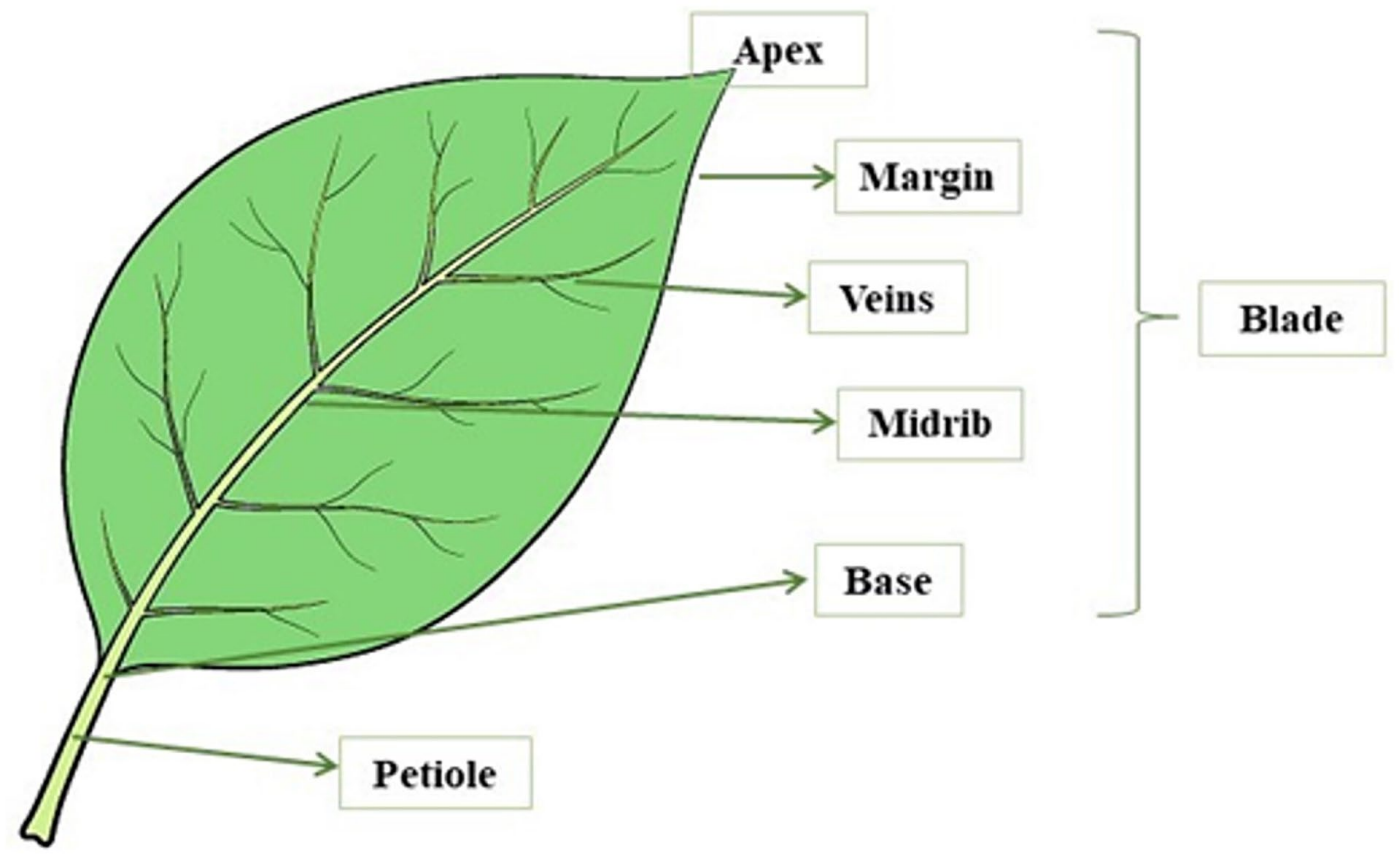
Leaf structure.

Leaves are photosynthetically active plant organs capable of storing absorbed solar energy in reduced organic compounds. These assimilated compounds represent a pool of energy and compounds used by the plant to meet the requirements for growth and development (101). A leaf consists of two main parts: the petiole (the stem that connects the leaf blade to the stem of the plant) and the blade (the widest portion of the leaf) (Figure 2).

The base is the area of the blade connected to the petiole, a stem-like structure that connects the leaf blade to the stem of the plant. The leaf blade is formed by several layers of cells, with the plant cells being relatively large and surrounded by a cell wall. In this area, a vein called a midrib runs through the center of the leaf. Leaf cells are filled with chloroplasts which are the organelles where photosynthesis takes place and contain specialized pigments defined as chlorophylls. These pigments allow plants to defend and capture energy from the sun and give the leaves their typical green color. Leaves have a waxy outer layer with stomata that are pores on the surface of the plant tissue and allow gas exchange with the environment. Leaves must maintain a balance between opening these pores to allow gas exchange and closing the pores to prevent water loss. Leaves have veins that completely cross the leaf structure and extend throughout the plant. These veins consist of structural vascular tissue and carry nutrients and water to all parts of the plant. Leaves also have other tissues with different functions, such as the mesophyll, which is the photosynthetic tissue, and the epidermis, which represents the outer layer of leaf cells. In addition, leaves vary greatly in size, shape, and various other characteristics such as blade edge character and vein type (102). Leaves are generally divided into two types: (i) Compound leaves consisting of several lobes; and (ii) Simple leaves made up of a single blade (103). One of the main functions of leaves is the photosynthetic production of starch, a source of chemical energy for plants. Photosynthesis is the process in plants that converts light energy into usable chemical energy (102), literally meaning “synthesis through the light” (104). In this pathway, the carbon dioxide and the water are absorbed by the environment and they are used to form sugars, oxygen, water, and chemical energy in the form of adenosine triphosphate (ATP). The formation of these compounds takes place in the presence of light energy and chlorophyll. This pigment, which is contained in the chloroplasts, is present in one-fifth of mesophyll in the inner tissue (parenchyma) of a leaf, and is responsible for the absorption of sunlight (102, 105).

Photosynthesis converts carbon from carbon dioxide in plants into organic compounds. This allows plants to produce organic building blocks from nucleic acids, amino acids, sugars, fatty acids, new cells, proteins, DNA and RNA, starches, vitamins and hormones, and many secondary compounds (106). The oxygen released by green leaves replaces the oxygen removed from the atmosphere during the respiration of plants and animals and by combustion. Oxygen is released into the atmosphere through stomata on the leaf surface, and hydrogen obtained from water combines with carbon dioxide in the enzymatic process of photosynthesis to form sugars (102).

Another important function of leaves is certainly transpiration, which is defined as the physiological loss of water in the form of water vapor, primarily through evaporation from leaf stomata, and the surface of leaves, flowers, and stems (107). Stomatal openings are necessary for carbon dioxide to enter the leaf and oxygen to be expelled during photosynthesis. It has been suggested that transpiration provides the energy to transport water within the plant and may help dissipate heat in direct sunlight (through cooling by water evaporation). About 97–99% of water absorbed by the roots is lost in this process and released into the air in the form of water vapor. Leaf stomata are the main site of transpiration and are composed of two guard cells that form small pores on the leaf surface. Guard cells can control the opening and closing of stomata in response to various environmental stimuli and regulate the rate of transpiration to reduce water loss. On the one hand, lighting, adequate water supply, and optimal temperature will open the stomata and increase transpiration. On the other hand, darkness and lack of water in the plant close the stomata and reduce transpiration. Many plants close their stomata in hot conditions to reduce evaporation, or at high concentrations of carbon dioxide when the plants appear to have enough for photosynthesis (102). In addition, various biochemical and morphological characteristics of plants can affect transpiration rates, such as leaf area, shape, and orientation, leaf surface features (such as the thickness of the cuticle and presence of hairs), root-shoot ratio, plant hormones, and age (108).

The main constituents of leaves are lignin, proteins (nitrogen), cellulose, hemicellulose, starch, chlorophyll, and water (109). These components are large chemical molecules that “trap” many mineral elements that are fundamental for plant life. The accumulation of minerals in plants is largely affected by genetic variation and the growth environment. Potassium (K), nitrogen (N), phosphorus (P), magnesium (Mg), sulfur (S), calcium (Ca), zinc (Zn), copper (Cu), and manganese (Mn) are some minerals present in plant leaves and their concentration changes according to the type of leaf (110–112). In plants, primary metabolites (carbohydrates, amino acids, organic acids) are important compounds for plant life and are directly involved in plant growth and development. Although not directly involved in the basic functions of growth, reproduction, and development of an organism, plants produce SMs that are essential for long-term survival and are involved in many functions, including defense against predators and attracting pollinators (113).

## 3 Beneficial health effects of popular and exotic fruit plant leaves

In the following chapters, some of the principal activities that leaves exert in the human body, mainly antioxidant, antimicrobial, and anticancer activities, especially those of popular and exotic fruit plants, have been summarized (Table 1). Furthermore, the main uses of leaves in traditional and folk medicine are indicated (Table 2). The main polyphenols in different fruit plant leaves are also reported in Table 3. Most of these studies present activities related to the presence of phenolic compounds.

**Table 1:** Biological potential of fruit plants leaves.

Plant species	Biological potential
Lingonberry (*Vaccinium vitis-idaea*)	Antioxidant and antitumor activity (100)
Chokeberry (*Aronia* spp.)	Cytotoxic activity against three malignant cell lines (SK-Hep1, HeLa, LS-174 T, and A-549 cells) (114, 115) Antileukemic action against the HL60 cell line (96) Antifungal and antibacterial activity (114) Reparatory action in female New Zealand rabbits with skin injury (116) Prevention of symptoms of proteins and lipids peroxidation and oxidative stress in rats brain (117) Reduction of blood glucose levels in animals with diabetes mellitus, and activation of glucose use by L929 and PC12 cell lines (83, 118)
Raspberry (*Rubus idaeus*)	Cytotoxic effect on HL60, Hep2, and SW480 lines (96, 119) Inhibitory effects on *L. monocytogenes* (120) Antithrombotic activity, reduction in blood glucose and cholesterol levels, bacterial growth suppression, and anticoagulant efficacy (121, 122)
Blackcurrant (*Ribes nigrum*)	Antimicrobial activity against *Listeria monocytogenes, Bacillus cereus, Yersinia ruckeri, Campylobacter jejuni, Proteus vulgaris, Candida albicans,* and *Aspergillus niger* (123, 124) Anti-inflammatory action on carrageenan-induced rat paw oedema (125, 126)
Cloudberry (*Rubus chamaemorus*)	Antibacterial activity against *Bacillus subtilis* (95)
Grape (*Vitis* spp.)	Protective action on blood capillaries and hemostasis (127) Photoprotective against ultraviolet (UV) damage (128, 129) Prevent and combat skin aging (130, 131) Antiproliferative properties on various cancer cell lines (e.g., MCF-7, HepG2) (99) Strong antimicrobial and radical scavenging activity against Gram-positive bacteria, Gram-negative strains, and yeast (132)
Water Pink Apple (*Syzygium aqueum, S. guineense, S. samarangense*)	Citotoxicity against cancer cell lines (MCF-7, HeLa, SiHa) (133, 134) Antimicrobial and antibacterial effects (135–138) Antidiabetic activity (139) Anti-inflammatory activity (140)
Hardy kiwi (*Actinidia arguta*)	Anti-allergic (141) Anti-inflammatory (142) Antioxidant (143) Antidiabetic (144–146) Antibacterial action against *Staphylococcus aureus* (147) Utilized in the treatment of inflammation-related disorders, specifically gout (148)
Sea buckthorn (*Hippophae rhamnoides* L.)	Antioxidant and immunomodulatory properties (149–154) Anti-inflammatory activity (155–157) Hepatoprotective activity (158–162) Adaptogenic properties (162–164) Improve cardiovascular health and manage metabolic issues related to obesity and insulin resistance (162, 165–168) Potent antimicrobial and anti-viral activity against Influenza, *Herpes* viruses, HIV, Dengue (169, 170) Antibacterial activity against *Bacillus cereus*, *Pseudomonas aeruginosa*, *Staphylococcus aureus*, and *Enterococcus faecalis* (171) Radioprotective effects (172–174) Wound-healing properties (162, 175)
Strawberry tree (*Arbutus unedo* L.)	Antimicrobial activity and antifungal activity (93)
Figs (*Ficus* spp.)	Antibacterial and antiviral activity (92, 176, 177)
Guava (*Psidium guajava*)	Antioxidant, antihypertensive, antidiabetic, antidiarrheals, anticancer, antimicrobial, lipid-lowering, and hepatoprotective effects (178, 179) Antifungal and antibacterial activity (180, 181) Larvicidal activity against *Chaoborus plumicornis* as well as marginal nematicidal (*Caenorhabditis elegans*) and insecticidal (*Drosophila melanogaster*) activities (84)
Loquat (*Eriobotrya japonica* L.)	Anti-inflammatory effects (182–186) Stronger antioxidant capabilities than 54 other medicinal plants (187) Hepatoprotective benefits against ethanol-induced toxicity (188) Prevention of the death of PC12 neuronal cells (189) Prevention and management of type-1 and type-2 diabetes (186) Hypoglycemic impact by reducing blood glucose levels (190) Reduced the rise in blood glucose, total cholesterol, and triglyceride levels (191) Showed high cytotoxicity in several cancer cell lines (182, 189, 192–195)
Mango (*Mangifera indica* L.)	Anti-tumor, antipyretic, antidiarrheal, antiparasitic, antifungal, anti-obesity, hypocholesterolemic, antiallergic, immunomodulatory, antiviral, antimicrobial, anti-inflammatory, antidiabetic, and antioxidant activities (82, 196, 197)
Papaya (*Carica papaya*)	Antitumor activity (98)
Pomelo (*Citrus grandis, C. maxima*)	Antibacterial activity against *Staphylococcus aureus*, *Candida albicans*, *Escherichia coli*, and *Pseudomonas aeruginosa* (198) *C. maxima* leaves have demonstrated positive effects in regulating blood glucose levels and reducing lipid peroxidation (199)
Kaffir lime (*Citrus hystrix, C. microcarpa*)	Antibacterial, anti-inflammatory, hepatoprotective, and antioxidant properties. Anti-tumor effect cervical cancer (HeLa) and neuroblastoma (UKF-NB3, IMR-5, SK-N-AS parental) cell lines. Tyrosinase inhibitory activity, antifertility, cardioprotective, and hepatoprotective, effects, larvicidal effects against *Aedes aegypti* (200, 201). *C. macrocarpa* shows strong inhibitory effects against bacteria (*Staphylococcus aureus, Pseudomonas aeruginosa, Escherichia coli,* and *Streptococcus mutans*) (202).
Citron (*Citrus medica*)	Cytotoxic effects on earthworms (203) Hypoglycemic potential (204) Estrogenic activity (205)
Lime (*Citrus aurantifolia*)	Antioxidant and anti-inflammatory properties (206) Antibacterial effects against Gram-positive (e.g., *Bacillus subtilis*) and Gram-negative (e.g., *Salmonella*, *Escherichia coli*) strains (207)
Bitter orange (*Citrus aurantium*)	Antibacterial activity against Gram-positive (*Staphylococcus aureus* and *Bacillus subtilis*) and Gram-negative bacteria (*Escherichia coli* and *Klebsiella pneumonia*) (208)
Sweet orange (*Citrus sinensis*)	Antibacterial activity against *Helicobacter pylori* (209) Induced inotropic depression on guinea pig atria (210) Increased bone mineral content and density, potentially aiding in osteoporosis management (211) Insecticidal activity against mosquito larvae (212) Strong bioactivity against various pests (213) Improved glucose control and liver glycogen levels (214)
Mandarin (*Citrus reticulata*)	Protect against alcohol-induced gastric ulcers (215) Anti-inflammatory characteristics (216) Can help prevent diseases associated with oxidative stress (e.g., type 2 diabetes and obesity) (217)
Grapefruit (*Citrus paradisi*)	Anti-inflammatory properties (218)
Lemon (*Citrus limon*)	Antibacterial properties against Gram-negative (e.g., *Salmonella typhimurium*, *Pseudomonas aeruginosa*) and Gram-positive (e.g., *Staphylococcus*) bacteria, and potential insecticides (207, 219)
Apple (*Malus domestica* Borkh.)	Antioxidative, antibacterial, anti-inflammatory, anticancer (against prostate cancer cells), and immunosuppressive potentials (37) Potential as an insecticide (220) Antifungal activity against *Penicillium expansum* (221) Antifeedant activity (222)
Wild apple (*Malus pumila*)	Antioxidant activity (223)
Quince (*Cydonia oblonga*)	Protective effects on spermatogenesis in hypercholesterolemia, anti-fungal properties, renoprotective potential, anti-atherogenic and hepatoprotective effects, anti-proliferative effects against colon cancer cells, and antioxidant potential (224–229)
Neem (*Azadirachta indica*)	Effective for anorexia and skin problems. Anti-inflammatory and antipyretic properties (230, 231)
Blackthorn (*Prunus spinosa*)	Antioxidant, antifungal (*P. ochrochloron, P. funiculosum,* and *T. viride*), and antibacterial (*B. cereus* and *E. cloacae*) activities (232) Antidiabetic activity (232) Anticancer effects on cell lines K562, MDA-MB-453, HeLa (232)
Peach (*Prunus persica*)	Stimulates gut activity (233) Anti-inflammatory, analgesic, and antipyretic effects (234) Cytotoxic effects against tumor cells (235, 236) Antiparasitic activity (237, 238) Anti-obesity effects (239–241) Anticholinesterase effects (242, 243) Antidiabetic effects (244–246) Protection against UV-induced damage (247–249)
Olive tree (*Olea europaea* L.)	Antimicrobial, and anti-inflammatory properties (250) Antibacterial activity against various bacteria such as *Escherichia coli*, *Klebsiella pneumoniae*, and *Staphylococcus aureus* (94, 251–253) Antioxidant and anticancer effects (254) Neuroprotective effects (255) Positive effect on the cardiovascular system (256–262)

**Table 2:** Traditional uses of some leaves from fruit plants.

Fruit	Medicinal uses
Blueberry (*Vaccinium angustifolium, Vaccinium myrtilloides*)	Leaves utilized for rheumatism (263) A tea from the leaves is utilized for women after abortion and to treat colic in infants (264, 265)
Lingonberry (*Vaccinium vitis-idaea*)	Preparation from leaves used to treat rheumatism, gout, and bladder problems (266)
Strawberry (*Fragaria virginiana*)	Leaves are used as a general tonic and for gargle to strengthen the gums, to treat urinary tract infections, bowel weakness, dysentery, and stomach cramps (263)
Black currant (*Ribes nigrum*)	Used to make infusions for the elimination of toxins from the body and regulation of renal function (125). Extracts from leaves utilized to treat rheumatic disease, as well as diaphoretic and diuretic agents (267, 268)
Raspberry (*Rubus idaeus*)	Tea made from leaves used for diarrhea and colic treatment, while compresses and poultices made from the infusion of leaves have been used to cure skin conditions (269, 270)
Chokeberry (*Aronia* spp.)	Mental and bone-healing elixir of youth, commonly given to expectant young women to enhance strength (269). Frequently used to function as anti-inflammatory, antiviral, antibacterial, and anti-proliferative agents (114).
Grape (*Vitis* spp.)	Crushed leaves of wild grapevines have been used for wounds and heavy menstruation and the dried leaves were grown to be snorted to stop nosebleeds. Leaf extract has been used to reduce leg swelling in the elderly. Boiled water with leaves is good for the liver (271) Crushed leaves of domestic vines and their extracts (infusion, decoction) were used especially in dysentery with bloody stools, but also in chronic diarrhea, bleeding and gastroenteritis, and venous circulatory conditions (127, 272, 273)
Water Pink Apple (*Syzygium aqueum*)	Leaves used in digestive disorders, liver detoxification, headaches, treatments for fever, skin diseases, and cancer prevention (274–276) The fresh leaves are used to relieve the pains of childbirth and in their dry powder form also to treat mouth ulcers (277)
Hardy kiwi (*Actinidia arguta*)	Leaves have been used to treat several inflammation-related disorders, including gastritis, pneumonia, and arthritis (142, 278, 279)
Pomegranate (*Punica granatum*)	Leaves have been employed to regulate inflammation and address digestive system problems (280, 281)
Guava (*Psidium guajava*)	Leaves are applied to ulcers, wounds, and for rheumatic pains, and they relieve toothache when chewed (282) Waterleaf extract is utilized to decrease the glucose level in diabetics’ blood (283) Leaves decoction is used to treat diarrhea, and stomach pain due to indigestion, and to do vaginal and uterine wash (284–287) The leaf is traditionally used in folk medicine to control and/or treat some diseases, as hypertension and diabetes mellitus (288, 289) Leaves powder is used in the treatment of cholera, epilepsy, and rheumatism, and leaf tincture is given to children suffering from seizures (276)
Loquat (*Eriobotrya japonica* L.)	Leaves have been used to treat various diseases like inflammation, cough, pain, asthma, chronic bronchitis, and diabetes (290)
Mango (*Mangifera indica* L.)	Leaves are used in traditional folk tea recipes as well as in the treatment of respiratory and diabetic conditions throughout Asia and Africa (291, 292)
Quince (*Cydonia oblonga*)	Leaves are commonly used in decoctions to alleviate nervousness, dysuria, insomnia, cough, cold, abdominal cramps, diarrhea, fever, and hyperglycemia (293–296)
Neem (*Azadirachta indica*)	Leaves are used for the treatment of human ailments (e.g., malaria) and pest control due to its insecticidal properties (220) Leaves are effective for anorexia and skin problems, and have anti-inflammatory and antipyretic properties (230, 231)
Blackthorn (*Prunus spinosa*)	Leaves are used in folk medicine as anti-septic, anti-inflammatory (232), digestive, diuretic, purgative, and astringent agents (297, 298), as well as a mild laxative (299)
Peach (*Prunus persica*)	Leaves served various purposes, functioning as anthelmintic, insecticidal, laxative, sedative, and vermicidal agents, and were also used to address conditions like piles, leucoderma, and whooping cough (300)

**Table 3:** Main polyphenols in different fruit plant leaves.

Phenol class	Phenolic compounds	Amount	Fruit plant leaves
Flavonoids	Epicatechin	0.37 mg/g	Apple leaves (223, 301, 302)
	Phloridzin	24.43–66.1 mg/g	
	Phloretin	0.15 mg/g	
	Phloretin-2’xylo-glucoside	n.q.	
	Quercitrin	2.06 mg/g	
	Isoquercitrin	8.4 mg/g	
	Procyanidin C1	n.q.	
	Procyanidin B2	n.q.	
	Procyanidin B1	n.q.	
	Quercetin-3-O-rutinoside	n.q.	
	Quercetin-3-O-galactoside	n.q.	
	Quercetin-3-O-rhamnoside	28.5 mg/g	
	Quercetin-3-O-arabinoside	10.7 mg/g	
	Quercetin-3-O-xyloside	9.5 mg/g	
	Quercetin-3-O-glucoside	1.55 mg/g	
		
	trans-caftaric acid	1.94–8.46 mg/g	Grape leaves (303)
	Quercetin-glucuronide	6.19–18.52 mg/g	
	Quercetin-glucoside	0.73–7.35 mg/g	
	Quercetin-rutinoside	0.13–1.65 mg/g	
	Quercetin-galactoside	0.13–1.81 mg/g	
	Kaempferol-glucuronide	0.24–1.69 mg/g	
	Kaempferol-glucoside	0.11–1.76 mg/g	
	Kaempferol-rutinoside	0.07–0.73 mg/g	
		
	P-hydroxybenzoic acid	2.15–3.76 mg/g	Ficus leaves (304)
	Sinapic acid glucoside	0.54–1.14 mg/g	
	Gallic acid derivative	1.06–1.61 mg/g	
	Caffeic acid derivative	1.32–2.64 mg/g	
	Coumaric acid derivative	0.89–1.21 mg/g	
	Caffeic acid	1.59–2.59 mg/g	
	Pro-anthocyanidin B1	0.54–0.93 mg/g	
	Caftaric acid	36.0–43.7 mg/g	
	Courmaroyl-hexose	0.32–9.10 mg/g	
	Quercetin −3,7-diglucoside	9.58–13.9 mg/g	
	Quercetin −3-triglucoside	1.74–9.88 mg/g	
	Cichoric acid	1.32–1.71 mg/g	
	Quercetin-3-glucoside	3.48–7.12 mg/g	
	Kaempferol-3-O-soporotrioside	0.16–1.11 mg/g	
		
	Catechin	n.q.	Cranberry leaves (302)
	Epicatechin	n.q.	
	Procyanidin B1	n.q.	
	Methoxyquercetin-pentoside	n.q.	
	Dimethoxymyricetin-hexoside	n.q.	
	Methoxyquercetin-3-O-galactoside	n.q.	
	Quercetin-3-O-rhamnoside	n.q.	
	Quercetin-3-O-galactoside	n.q.	
	Myricetin-3-xylopiranoside	n.q.	
		
	Rutin	3.16–6.14 mg/g	Other berry leaves (91, 270)
	Myricetin	< 2.74 mg/g	
	Myricetin-malonylglucoside	0.042–0.055 mg/g	
	Kaempferol-3-glucoside	0.017–4.78 mg/g	
	Kaempferol-3-O-rutinoside	0.019–0.036 mg/g	
	Kaempferol-3-O-malonylglucoside	0.135–0.409 mg/g	
	Kaempferol-3-O-glucuronide	9.23 mg/g	
	Kaempferol	0.171–0.244 mg/g	
	Luteolin-3-glucoside	< 1.10 mg/g	
	Epicatechin	1.27–4.07 mg/g	
	Catechin	0.72–7.89 mg/g	
	Quercetin-3-glucoside	0.038–10.44 mg/g	
	Quercetin-3-O-galactoside	0.057–0.081 mg/g	
	Quercetin-3-O-rutinoside	0.099–0.229 mg/g	
	Quercetin-3-O-malonylglucoside	2.424–3.890 mg/g	
	Quercetin-3-O-glucuronide	7 mg/g	
	Quercetin	< 10.369 mg/g	
	Isoquercetin	4.60 mg/g	
	Quercetrin	19.47 mg/g	
	Procyanidin B2	< 3.38 mg/g	
	Sanguiin H-6	< 73.92 mg/g	
	Lambertianin C	16.75–123.41 mg/g	
	Casuarinin	34.47–117.86 mg/g	
	Hyperoside	12.09 mg/g	
		
	Rutin	9.3 mg/g	Peach leaves (305)
	Kaempferol-3-O-rutinoside	4.057 mg/g	
	Kaempferol-3-O-glucoside	20.112 mg/g	
	Kaempferol-3-O-rhamnoside	1.296 mg/g	
	Quercetin	6.954 mg/g	
	Kaempferol	12.393 mg/g	
Phenolic acids	Chlorogenic acid	n.q.	Apple leaves (302)
	Neochlorogenic acid	n.q.	
	p-coumaric-quinic acid	n.q.	
	Cryptochlorogenic acid	n.q.	
		
	p-coumaric acid	0.490–6.007 mg/g	Berry leaves (91, 270)
	Ferulic acid	7.537–19.87 mg/g	
	Chlorogenic acid	0.081–61.31 mg/g	
	Caffeic acid	< 0.55 mg/g	
	Ellagic acid	3.31–4.48 mg/g	
	Gallic acid	0.18–1.76 mg/g	
	Neo-chlorogenic acid	0.044–0.435 mg/g	
		
	Caffeic acid derivatives	2.497 mg/g	Peach leaves (305)
	p-Coumaric acid derivatives	1.018 mg/g	
	Chlorogenic acid	4.785 mg/g	
	Caffeic acid	0.635 mg/g	

n.q.: not quantified—compounds were only qualitatively described.

### 3.1 Berry fruits

#### 3.1.1 *Vaccinium* spp., *Fragaria* spp., *Ribes* spp., *Rubus* spp., *Aronia* spp.

Indigenous people used fruit leaves of berry species as food and medicinal plants, such as labrador tea and bog rosemary, but also strawberries (*Fragaria virginiana*), lingonberries (*Vaccinium vitis-idaea*), and blueberries (*Vaccinium angustifolium, Vaccinium myrtilloides*) (306). The roots and leaves of low-bush blueberries have been used to treat rheumatoid arthritis: they are boiled, and the resulting liquid is applied to painful areas of the body (263). In addition, tea made from the leaves was consumed for its anti-inflammatory properties. It has been used to treat colic in babies and has also been given to women after an abortion. It is very interesting to know that the blueberry plant is now well-documented to contain powerful anti-inflammatory compounds such as polyphenols (264, 265). Another berry, whose leaves are used in abundance, is the lingonberry (*Vaccinium vitis-idaea*). The plant grows in boreal, northern temperate, and subarctic climates, and produces small red berries (307). Historically, this species has been a popular wild fruit widely cultivated and consumed in Scandinavian countries but has increased in popularity in North America in recent years (307, 308). Many indigenous peoples of North America and Eurasia have used lingonberry leaves and fruits for food and medicine (309). Preparations made from the leaves have been used to treat rheumatism, gout, and bladder problems (266). Lingonberries have been extensively studied over the past few years and are now known to contain several bioactive polyphenols (e.g., quercetin, anthocyanins, proanthocyanidins, and resveratrol) that have beneficial effects against cardiovascular disease (CVD), anti-cancer properties, and inhibition of neurodegeneration (307, 310–312). In addition, lingonberry leaf extracts have been shown to have a protective effect on brain cells, possibly due to their anti-inflammatory and antioxidant properties (313). Another example of a berry used by indigenous people is the strawberry (*Fragaria virginiana*). They chewed or soaked the leaves in water to make tea to treat stomach cramps. The strawberry leaves can be also used as a natural tonic, employed to strengthen and gargle the gums, to treat urinary tract infections, bowel weakness, and dysentery (263). *Ribes nigrum* (black currant) leaves were used to make infusions that had the effect of accelerating the elimination of toxins from the body and regulating renal function (125). Extracts from black currant leaves have also been utilized to treat rheumatic disease, as well as diaphoretic and diuretic agents (267, 268). For ages, diarrhea and colic have been treated with tea made from *Rubus idaeus* L. (raspberry) leaves, while compresses and poultices made from the infusion of raspberry leaves have been used to cure skin conditions (269, 270). Native Americans in the United States and people in Siberia employed the leaves of *Aronia* (chokeberry) as a mental and bone-healing elixir of youth. *Aronia* leaves were also commonly given to expectant young women due to their ability to enhance strength (269). Moreover, *Aronia* leaves were frequently used in folk medicine to function as anti-inflammatory, antiviral, antibacterial, and anti-proliferative agents (114). *R. chingii* leaves were frequently utilized in China to make famous drinks like tea. According to reports, *R. chingii* intake has positive benefits on health, which are mostly attributed to their antioxidant properties, mainly due to the presence of flavonoids (kaempferol, quercetin, tiliroside, quercetin 3-O-b-D-glucopyranoside, and kaempferol 3-O-b-D-glucopyranoside) (121, 314–316). However, little research has been done to determine how the active compounds of *R. chingii*, particularly the polysaccharides, relate to its physiological effects.

Today, these traditional uses are largely forgotten. In the last decade, the European Medicines Agency (EMA) has approved the sale of infusions and extracts from leaves of several fruits such as *Ribes nigrum*, *Rubus idaea,* and *Arctostaphylos uva-ursi* as herbal medicinals based on their traditional use (270, 317–319). More recently, also the sale of leaf extracts of strawberries (*Fragaria* spp.) for the same use has been approved (320).

It can be said that the pattern of phenolic compounds and the fact that each species has a particular profile of these compounds, is primarily responsible for the beneficial effects of berry leaf extracts. For instance, in blackcurrant leaves, the main phenolic compound is represented by *p*-coumaric acid, followed by neochlorogenic, cryptochlorogenic, and chlorogenic acids (123, 267, 302, 321, 322). Only bilberry leaves had more chlorogenic acid than blackcurrant leaves, which also had more of this substance than strawberry and raspberry leaves. Blackcurrant also has the highest concentration of quercetin-3-O-derivatives, with quercetin-3-O-glucosyl-6′-acetate being the most prevalent (321). Kaempferol, myricetin, and their derivatives were among the other flavonoids found in blackcurrant leaves (268). In *Rubus* leaves, the most abundant compounds are quercetin-3-O-derivatives (mostly quercetin-3-O-glucuronide) and caffeic, ellagic, and p-coumaric acid, which are more than other berry leaves such as strawberry and blueberry (91, 96, 323–326). Cloudberry (*Rubus chamaemorus* L.) leaves contain various bioactive compounds such as gallic acid, ellagic acid, flavonoids, and their derivatives (95). Strawberry leaves are notable for having a high amount of gallic acid (96). *Aronia* leaves have the highest levels of sinapic acid (114), with other phenolic compounds like chlorogenic, neochlorogenic, 3,4-dihydroxyphenylacetic, and protocatechuic acids being present (96, 327). Similar to blackcurrant and raspberry, quercetin and its derivatives are the main flavonoids found in *Aronia* leaves (91, 114, 327). Red currant leaves also have high amounts of chlorogenic acid (91). Even the leaves and fruits of *Vaccinium vitis-idaea* L. (lingonberry) are complex matrices containing phenolic compounds with different structural characteristics. Lingonberry leaf extract is characterized by a high content of flavonols, some proanthocyanidins, catechins, and arbutin (100).

In general, berry leaves contain more phenolic chemicals than the same species’ fruits do. According to Tabart et al. (268) and Teleszko and Wojdyło (302), the phenolic patterns of the leaves of raspberry, black currant, and *Aronia* plants differ from those of the corresponding berries. Recently, different studies found that raspberry, blackberry, and strawberry leaves have a higher antioxidant capacity than fruit and that they are used as excellent low-cost sources of bioactive compounds with potential for industrial use (323, 328–330). Cvetanovic et al. (114) demonstrated that *Aronia melanocarpa* leaf extracts had a higher biological potential and chemical composition than extracts from stems and berries, as well as having the highest concentrations of total phenolic compounds and flavonoids. Other scientists showed that, depending on many variables, it is also feasible to affect the concentration of phenolic chemicals in berry leaf extracts. Paunovic et al. (123) highlighted that soil management systems can influence the amount of anthocyanin in black currant leaves, as well as the synthesis and accumulation of both flavan-3-ols and flavonols in berries and leaves. Nour et al. (267) studied the antioxidant activity and the content of phenolic compounds in six black currant cultivar leaves at different times, finding that leaves collected in mid-June presented the highest total phenolic content and antioxidant capacity. Also, a study demonstrated that younger leaves showed a greater antioxidant activity than those gathered at later stages of growth (115). Furthermore, the highest concentration of total phenols in black currant leaves was found to be at the end of August, although the content of several phenolic compounds was greatest in June (322). Additionally, *Aronia* leaves collected in late season (September) had a greater amount of total phenolics and antiradical activity compared to those collected in earlier stages (July) (327). Lastly, Cvetkovic et al. (331) observed that in *Aronia* leaves gathered in the senescence phase (November), the total content of phenolic compounds was still high, indicating that the synthesis of phenolic compounds occurs during the entire growing season.

Phenolic compounds with high antioxidant capacity may interfere with ROS-mediated carcinogenesis. This was confirmed by new data on the viability of cancer cells, reduced by the activity of basic phenolic compounds in lingonberries. Among them, quercetin and proanthocyanidins, as well as a promising chemical activator for new herbal preparations and nutritional supplements, can be considered markers of the antioxidant and antitumor activity of lingonberry leaf and fruit extracts (100). The cytotoxic activity of *Aronia* leaf extracts against several cancer cell lines was one of the subjects of most research. Due to the abundance of flavonoids, which are primarily responsible for the cytotoxic impact, *Aronia* leaves extract, for instance, showed stronger cytotoxicity than extracts from other plant components against three malignant cell lines (HeLa, LS-174 T, and A-549 cells) (114). The hydrolyzed extract of *Aronia* leaves inhibited the development of the multidrug-resistant sublines HL60/VINC and HL60/DOX and also exhibited antileukemic action against the HL60 cell line (96). Concerning SK-Hep1 human hepatoma cells, *Aronia* leaf extract also showed anticancer activity, inhibiting metastasis, and cell growth in a dose-dependent manner (115). Similarly, raspberry leaf extract has been observed to have a cytotoxic effect on the sensitive leukemia HL60 line (96). It was also found that compounds such as quercetin, ellagic acid, and gallic acid are associated with cell growth inhibition. Furthermore, raspberry leaf extract has also been reported to have a cytotoxic effect on human laryngeal carcinoma (Hep2) and colon adenocarcinoma (SW480) cell lines, with the latter being more susceptible (119).

Leaves of some berry species were tested for possible antimicrobial activity. In the Milenkovic-Andjelkovic et al. (91) research different species of berries have been analyzed: wild species such as Blackberry (*Rubus fruticosus*), Blackthorn (*Prunus spinosa*), Hawthorn (*Crataegus L.*), Dog rose (*Rosa canina*), and European cornel (*Cornus mas*), and domestic species such as Blackberry (*Rubus fruticosus*), Raspberry (*Rubus idaeus*), Black Currant (*Ribes nigrum*), and Red Currant (*Ribes rubrum*). The antimicrobial activity of these samples was evaluated using the following control strains: yeasts such as *Candida albicans* ATCC 10231, Gram− bacteria such as *Proteus vulgaris* ATCC 8427, *Klebsiella pneumoniae* ATCC 10031, *Shigella sonnei* ATCC 25931, *Salmonella enteritidis* ATCC 13076, *Pseudomonas aeruginosa* ATCC 9027, *Escherichia coli* ATCC 25922, and Gram+ bacteria such as *Micrococcus flavus* ATCC 40240, *Sarcina lutea* ATCC 9341, *Staphylococcus aureus* ATCC 8538, *Listeria monocytogenes* ATCC 7644, *Bacillus cereus* ATCC 8739, and *Clostridium perfringens* ATCC 19404. In all tested leaf extracts, among other bioactive compounds, phenolic acids such as gallic, ellagic, and chlorogenic acids, are responsible for important antimicrobial and antioxidant actions. The studied leaf extracts were less effective against Gram− strains, and yeasts compared to Gram+ strains. The most susceptible Gram+ strains were *Staphylococcus aureus*, *Listeria monocytogenes,* and *Sarcina lutea*. On the other hand, *Shigella sonnei* and *Pseudomonas aeruginosa* are the most sensitive Gram− strains for the most investigated leaf extracts (91). Tian et al. (120) verified that the chemicals found in raspberry leaves have inhibitory effects on *L. monocytogenes*. The antimicrobial activity of blackcurrant leaf extracts against *Listeria monocytogenes*, *Bacillus cereus*, *Yersinia ruckeri*, *Campylobacter jejuni*, *Proteus vulgaris*, *Candida albicans*, and *Aspergillus niger* was investigated in other studies by Paunovic et al. (123) and Raudsepp et al. (124). *Aronia* leaf extracts were also tested for antifungal and antibacterial activity against two fungal species, four gram-negative, and two gram-positive bacterial strains. These tests revealed that the *Aronia* leaf extracts had antifungal and antibacterial activity that was 15 times stronger than the antibiotic amracin against *Proteus mirabilis* and four times stronger against *P. vulgaris*. In addition, the antifungal activity produced results that were comparable to those of the antifungal drug nystatin (114). The extract of *R. chamaemorus* leaves was tested on various Gram− and Gram+ bacteria and it was demonstrated that the leaf extract was active only against some Gram-positive bacteria such as *Bacillus subtilis* (95). The antibacterial effects of leaf extracts were found to be linked to the presence of phenolic compounds. Numerous authors reported that the glycosylation of flavonols may influence their growth inhibitory capacity and that the number of hydroxyl groups in the phenolic compounds may influence their activity against bacteria (332, 333). The antibacterial capacity of phenolic acids has also been reported to be related to the presence of the carboxyl group and the substitution pattern in the benzene ring (334).

Proinflammatory substances such as TNF-α, IL-1, and CINC-1 were able to be reduced by water-alcoholic black currant leaf extracts on carrageenan-induced rat paw edema. Niflumic acid and indomethacin were used as benchmarks; their anti-inflammatory action was comparable to that, but they lacked ulcerative potential (125, 126). Extracts from raspberry leaves demonstrated antithrombotic activity, a reduction in blood glucose and cholesterol levels, bacterial growth suppression, and anticoagulant efficacy (121, 122). After seven days of treatment, *Aronia* leaf extracts completely epithelialized the tissue and reduced erythema and edema in female New Zealand rabbits with skin injury, demonstrating their reparatory action (116). Assessments both *in vitro* and *in vivo* also demonstrated the effect of *Aronia* leaves in preventing symptoms of proteins and lipids peroxidation and oxidative stress in rats’ brains (117). Intraperitoneal or oral administration of *Aronia* leaf extract had a meaningful effect on reducing blood glucose levels in healthy rats and animals with diabetes mellitus induced by streptozotocin, while extracts from *Aronia* leaves were shown to activate glucose use by L929 and PC12 cell lines (83, 118).

In addition to phenolic chemicals, berry plants’ leaves also include trace elements including copper (Cu), zinc (Zn), manganese (Mn), and boron (B), as well as minerals like potassium (K), calcium (Ca), magnesium (Mg), phosphorus (P), sodium (Na), and iron (Fe) (331). Black currant leaves had the largest concentration of Ca, Mg, P, and Fe, whereas raspberry leaves largely contained Fe, Mn, Ca, K, B, and Na, and chokeberry leaves had significant concentrations of Mn, Zn, and Fe. Growth conditions, cultivation method, abiotic and biotic stress, and nutritional status are the variables that might affect the concentration of minerals in berry leaves (335–338). Black currant leaves picked in mid-June, for instance, had increased concentrations of Ca, K, and Mg ions as well as Fe and Mn (267, 337). The trace elements and minerals indicated above play a significant part in the cellular antioxidant system’s metabolism (339). Iron and other microelements are crucial as components of the storage systems and oxygen transport and as enzyme cofactors (338, 340, 341), whereas K, Mg, and Ca have been linked to a decreased risk of stroke, hypertension, and osteoporosis (341). Due to its role as a structural element in the enzymes xanthine oxidase and dehydrogenase, molybdenum is essential for the production of urea (331).

#### 3.1.2 Grape (*Vitis* spp.)

Grape leaves have been used in folk medicine since classical Greek times and were common in isolated rural areas of the Iberian Peninsula until the middle of the last century (342). Crushed wild grape leaves have been used for heavy menstruation and wounds, and dried leaves have been crushed to be snorted to stop nosebleeds. The leaf extract resulting from the maceration of ground leaves in water has been used to reduce leg swelling in the elderly. Boiling water with leaves is good for the liver (271). All these uses are consistent with the known beneficial effects of domestic grape leaves, especially those of red cultivars, which contain astringent tannins, many flavonoid pigments (riboflavin), anthocyanins, various vitamins, and macro and micro elements such as calcium, copper, iron, manganese, and magnesium, which perform a protective action on blood capillaries and hemostasis (127). Crushed domestic grape leaves and their infusions and decoctions were used in the following cases: (i) the venous circulation conditions (272, 273); (ii) bleeding, especially useful during menopause, to prevent frequent blood loss; (iii) chronic diarrhea, gastroenteritis, and especially bloody dysentery (127).

Grapevine leaves are rich in antioxidants and other bioactive compounds. They contain a great concentration of vitamins A and K and other bioactive compounds such as anthocyanins, tannins, terpenoids, catechins, various acids (malic, silicic, citric, tartaric, and succinic acids), resveratrol, enzymes, lipids, and carbohydrates (343, 344). Studies have shown that grape leaves contain ten times more antioxidant properties than grape juice and pulp (345). The ‘Pinot noir’ leaves extract showed a strong antioxidant capacity and can be used as a photoprotective against ultraviolet (UV) damage (128, 129). Various studies have shown that grape leaves can be used to prevent and combat skin aging, which is associated with a gradual decrease in the ability of human skin cells to repair DNA damage due to the generation of free radicals (130). For example, Marabini et al. (131) demonstrated *in vitro* protective effects of polyphenolic compounds from grape leaves against UV-induced skin damage. Although many synthetic photoprotective compounds are available, they have potential toxicity, mainly due to ROS induction in human skin (346). Therefore, it is important to develop new strategies to prevent, reduce, or treat UV radiation damage using natural products: in this view, leaves possess a great potential for the pharmacological and cosmetic industry to be a candidate as a natural source for obtaining extracts with strong UV protection effect (347, 348).

The numerous phytochemical compounds in grape leaves attracted the interest of many researchers for their potential antiproliferative effect. A study by Ferhi et al. (99) analyzed the antiproliferative properties of polyphenols present in water and ethanol extracts of grape leaves on various cancer cell lines such as MCF-7 breast cancer cells, and HepG2 human hepatoma cells. The apoptotic processes underlying carcinogenesis are activated by two main pathways: death receptor-dependent and mitochondria-dependent apoptotic pathways. In mitochondria-dependent signaling pathways, the Bcl-2 protein family includes two groups: apoptosis activators (Bad, Hrk, Bok, and Bax) and apoptosis inhibitors (Mcl-1, Bcl-XL, and Bcl-2). The Bax/Bcl-2 ratio may be an important factor influencing cell behavior. Suppression of Bcl-2 promotes apoptosis in response to several stimuli, including anti-cancer drugs (349). Bax is an inactive cytoplasmic apoptotic protein. When Bax is activated, it moves into the mitochondria and plays an important role in mitochondria-mediated apoptosis. Activated Bax creates pores in the outer mitochondrial membrane, causing leakage of ions, major metabolites, and cytochrome c from the mitochondria into the cytosol, promoting cell death. In cells cultured with grape leaf extract, the mRNA level of the anti-apoptotic factor Bcl-2 was reduced, and the expression of the pro-apoptotic Bax gene was significantly induced. It was found that the crude ethanolic extract was able to induce a greater antiproliferative effect compared to the crude aqueous extract. This could be due to the different amounts of phenol present in water and ethanol extracts (99).

The extracts of two red grape cultivars (Vranac and Merlot) also showed strong antimicrobial and radical scavenging activity, mainly due to the presence of numerous phenolic compounds (flavonols, flavan-3-ols, phenolic acids, and stilbenes). The strongest antimicrobial activity was shown against Gram-positive bacteria (*Staphylococcus aureus, Sarcina lutea*, *Clostridium perfringens*, *Bacillus cereus*, *Listeria monocytogenes,* and *Micrococcus flavus*), followed by Gram-negative strains (*Escherichia coli*, *Salmonella enteritidis, Pseudomonas aeruginosa, Klebsiella pneumoniae,* and *Shigella sonnei*) and yeast (*Candida albicans*) (132).

#### 3.1.3 Watery pink apple (*Syzygium aqueum*)

The *Syzygium aqueum*, common name Watery Pink Apple, is widely used in folk medicine and is known for its many biological activities. *S. aqueum* is known to have several pharmacological effects, and the leaves appear to be the most exploited part. The fruit, bark, and leaves have many medicinal uses such as for liver detoxification, digestive disorders, headache, skin diseases, cancer prevention, and fever treatment (276) due to the presence of many phenolic compounds such as flavonoids, phenolic acids, anthocyanins, lignans, and tannins (140). In tropical Asia, it is used for several herbal applications (274, 275). In Malaysia, dried leaf powder is used to treat cracked tongues (276). The dried leaves are also eaten with vegetables and used to treat mouth ulcers and the raw fresh leaves are used to treat pneumonia and malaria and to relieve the pain of childbirth. Leaves infusion is used in the treatment of dysentery and stomach pains (277).

The radical scavenging activities of the methanolic extracts of *S. aqueum* fresh leaves were found to be similar to standard compounds [(epi) Gallocatechin gallate and vitamin C], displaying better results than the dried leaves (140). Meanwhile, the leaf extract of *S. cumini*, extracted with either ethanol or methanol, displayed a strong antioxidant capacity (350, 351). On the contrary, the methanolic extract of *S. guineense* leaves exhibited no antioxidant activity (139). However, the essential oils extracted by the hydro-distillation method showed a high antioxidant activity (138).

The cytotoxicity of the methanolic extract of *S. aqueum* leaves was evaluated on two different breast cancer cell lines (MCF-7 and MDA-MB-231). The extract was found to be less active on MDA-MB-231 cancer cells, while it demonstrated high cytotoxicity towards MCF-7 cells. This activity has been attributed to the presence of phenolic compounds acting as phytoestrogens in the studied *Syzygium* extract (134). Moreover, another study investigated the cytotoxicity of the leaf extracts of *S. guineense* in water, ethanol, and the mix of ethanol-water against the HeLa and SiHa cell lines. The results indicated that the ethanol extract was more effective in inhibiting both cell lines than the water and the ethanol-water leaf extracts (133).

The efficacy of ethanol leaf extract as an antibacterial was evaluated in *S. samarangense* samples. It was more effective against *S. enterica* and *B. cereus* than *Kocuria rhizophila*, *Enterobacter aerogenes*, and *E. coli* when compared with the standard antibacterial drug chloramphenicol (135). Another study checked the antibacterial activity of different ethanol and methanol leaf extracts of cultivars of *S. samarangense* against *S. aureus*, *B. cereus*, *P. aeruginosa*, and *E. coli*, with tetracycline as the positive control. All the extracts demonstrated antimicrobial action, with the ethanol extracts being more potent than the methanol extract (136). The methanol leaf extract from *S. cumini* was found to possess antibacterial activity towards *E. coli* and *S. aureus* (137). Essential oil from *S. guineense* leaves extract was effective against *C. albicans*, *Mycobacterium bovis*, *E. coli*, *S. aureus*, *P. aeruginosa*, and *K. pneumonia* when compared to standard antimicrobial drugs (ciprofloxacin, isoniazid, and fluconazole) (138).

*S. guineense* methanolic leaf extract displayed powerful antidiabetic activity (139). Furthermore, the anti-inflammatory activity of *S. aqueum* methanolic leaf extract was studied, gauging the capacity of the extract to impede inflammatory markers such as ovine COX-1 and COX-2, and lipoxygenase (LOX). The leaf extract presented a more robust inhibitory effect than the standard anti-inflammatory drug (diclofenac) on LOX and COX-2, and also greater than celecoxib on COX-1 (140).

#### 3.1.4 Hardy kiwi (*Actinidia arguta*)

*A. arguta* leaves are a traditional herbal remedy widely utilized in Asian nations (352, 353). High numbers of leaves are cut to maximize sun exposure throughout the hardy kiwi’s development (354). The leaves have been used to treat several inflammation-related disorders, including gastritis, pneumonia, and arthritis, in addition to their usage as a vegetable in Korea and China (142, 278, 279).

Hardy kiwi leaves have been cited as a prospective source of high added value chemicals, notably polyphenols (147, 354, 355). Previous studies reported an array of beneficial effects of this plant, such as anti-allergic (141), anti-inflammatory (142), and antioxidant (143) properties, as well as antidiabetic activity mainly attributed to its phenolic fraction (144–146). Both Ravipati et al. and Almeida et al. used water and ethanol as solvents in traditional extraction methods to recover polyphenols from *A. arguta* leaves (147, 353). Ravipati et al. (353) reported that the ethanolic extract of *A. arguta* leaves featured the highest TPC and TFC, while the aqueous extract had the greatest antioxidant activity. Based on its capacity to reduce hydrogen peroxide (H_2_O_2_)-induced yeast oxidative stress, the aqueous leaves extract showed suppression of yeast (*Saccharomyces cerevisiae*) oxidation (353). According to the authors’ hypothesis, the combination of trace metals and polyphenols significantly increased the antioxidant activity. Almeida et al. (147) examined the health-promoting effects of three distinct extracts (alcoholic, hydro-alcoholic, and aqueous) of *A. arguta* leaves. Notably, *A. arguta* leaf extracts demonstrated RNS and ROS scavenging activities (147). The alcoholic extract demonstrated the strongest antioxidant activity as determined by the FRAP and DPPH assays, most likely as a result of its greatest phenolic concentration, which was followed by the aqueous and hydro-alcoholic extracts. It also had the highest total flavonoid content (TFC) (147). The greatest scavenger of superoxide anion radicals was the aqueous extract, but the most efficient scavenger of hypochlorous acid (HOCl) and H_2_O_2_ was the alcoholic extract. Regarding RNS, alcoholic extract further showed the best scavenging effectiveness against peroxynitrite and nitric oxide radicals. But according to Almeida et al. (147), the synergistic impact of several compounds may help to boost the scavenging power. Based on this extract’s ability to scavenge hypochlorous acid, H_2_O_2_, and superoxide anion radical, an intriguing antioxidant activity was hypothesized. Hardy kiwi leaf extract also showed a potent capacity to quench peroxynitrite and nitric oxide radicals in the presence and absence of bicarbonate. A putative antibacterial action against *Staphylococcus aureus* was also seen in hydro-alcoholic and alcoholic extracts, which Almeida et al. (147) theorized may be due to the presence of phenolic acids and flavonoids. Recently, Marangi et al. (354) employed a new, green, and sustainable extraction technique, dubbed Multi-Frequency Multimode Modulated (MMM) technology, to remove bioactive compounds from *A. arguta* leaves using water as solvent. This extract demonstrated a higher TPC than the aqueous one obtained through conventional extraction by Almeida et al. (147) and also reported a high antioxidant activity through FRAP and DPPH assays. The extract was more efficient against peroxynitrite and hypochlorous acid. The flavonoid derivatives quercetin-3-O-(acetyl-rhamnoside)-hexoside and kaempferol-3-O-(acetyl-rhamnoside)-hexoside, as well as the chlorogenic acid derivatives (quinic acid, 3-CQA, and 5-CQA) may be primarily responsible for the extract’s ability to scavenge free radicals (354).

The erythrocyte membranes are effectively shielded from oxidation by free radicals brought on by physicochemical factors, such as chemical substance AAPH and ultraviolet radiation (UV) types B and C, according to Cyboran et al. (355) analysis of a methanol:water (50:50) extract from *A. arguta* leaves. The leaves extract showed higher antioxidant ability than butylated hydroxyanisole (BHA) and ascorbic acid, but lower than the major phenolic compounds present in the extract, namely procyanidin B2, procyanidin B3, catechin, and neochlorogenic acid. This extract showed an even higher antioxidant activity against free radicals induced by AAPH than by UVB and UVC. Heo et al. supported the traditional claims relating to the use of hardy kiwi leaves in the treatment of inflammation-related disorders, specifically gout (148). The results showed that the leaves inhibited NLRP3 inflammasome activation (which has a role in detrimental inflammatory syndromes) in both *in vitro* and *in vivo* models. In addition, HPLC fingerprinting identified rutin as the only polyphenol present, although it did not affect NLRP3 inflammasome activation, thus indicating that *A. arguta* leaves might have a synergistic effect on its attenuation (148, 356, 357). In particular, Ravipati et al. (353) claimed that hardy kiwi leaves have potent anti-inflammatory activities based on the efficient suppression of TNF-α production and down-regulation of NO production without impacting cell viability.

High-performance liquid chromatography (HPLC) analysis indicated that the predominant phenolic group in hydro-alcoholic and alcoholic extracts was flavan-3-ols (147). This can be attributed to the anticipated solubility of each compound in different solvents since phenolic acids were more soluble in aqueous solutions whereas flavan-3-ols were more easily extracted with alcohol (358, 359). However, compared to the alcoholic extract prepared by Almeida et al. (147), Cyboran et al. (355) reported a higher total amount of phenolic compounds for methanol:water extract (50:50). The different solvents used to draw out the phenolic compounds from leaves could explain the disparity between these results. Among the polyphenols identified, the most abundant were caffeoylquinic, cryptochlorogenic, neochlorogenic, and chlorogenic acids, glycosylated kaempferol and quercetin derivatives, catechin and B-type procyanidin dimers (354, 355). Based on the flavonoids present in the leaves, various *Actinidia* species were distinguished by Webby et al. (360) in an ethnobotanical investigation. The leaves of three different species of *A. arguta*, var. *purpurea*, *cordifolia*, and *arguta*, were combined into the *Leiocarpae* section and *Lamellatae* series, and their flavonoids profiles, including the presence of acylated compounds, showed great resemblance. Both *A. arguta* var. *arguta* and *purpurea* had low amounts of quercetin glycoside, although only the latter showed trace quantities of kaempferol and quercetin 3-O-xylogucoside. The presence of certain triglycosides was found in the three *A. arguta* types. However, only in two types (var. *arguta* and *purpurea*) 3-O-rhamno(1,6) glucosides/galactosides were found (360).

In addition to polyphenols, leaves also contain significant amounts of trace metals, which have been reported to be co-factors of antioxidant enzymes such as ascorbate peroxidase, superoxide dismutase, and other enzymes of the ascorbate-glutathione pathway. This suggests their involvement in antioxidant mechanisms (353, 361). Magnesium was the most abundant trace metal in the aqueous extract, while molybdenum and selenium had the lowest levels. Additionally, substantial amounts of manganese, copper, and zinc were determined (353).

#### 3.1.5 Sea buckthorn (*Hippophae rhamnoides* L.)

Sea buckthorn, scientifically known as *Hippophae rhamnoides* L. or *Elaeagnus rhamnoides* L., is a versatile plant deeply rooted in culinary and medicinal traditions. This “sea berry” stands out due to its rich amalgamation of bioactive compounds across its various parts, including flavonoids, phenolic acids, proanthocyanidins, carotenoids, fatty acids, triterpenoids, vitamins, and phytosterols, which collectively underpin its medicinal value (362). Research has primarily focused on sea buckthorn berries, unveiling a wide spectrum of potential health benefits (362). Turning to sea buckthorn leaves, they are equally nutrient-rich and brimming with bioactive substances. These leaves harbor flavonoids, carotenoids, sterols, triterpenols, and isoprenols, and serve as a substantial source of antioxidants like β-carotene, vitamin E, catechins, ellagic acid, ferulic acid, and folic acid. They also provide noteworthy amounts of essential minerals such as calcium, magnesium, and potassium. The polyphenolic compounds, including flavonols, leucoanthocyanidins, catechin, myricetin, and gallic acid, contribute to their antioxidant process (162, 363). Mineral analysis reveals their richness in calcium, magnesium, and potassium, with notable traces of sodium, manganese, iron, and zinc (161, 162, 363).

Sea buckthorn (SBT) has been the subject of extensive research into its antioxidant and immunomodulatory properties, with both *in vitro* and *in vivo* studies offering valuable insights into its potential therapeutic benefits. *In vitro* experiments using rat spleenocytes, macrophages, and the C-6 glioma cell line have yielded promising results. An alcoholic leaf extract of SBT demonstrated the ability to counteract chromium-induced free radical production, and apoptosis, and restore antioxidant levels and mitochondrial function to levels comparable to control cells (149). This extract also stimulated the production of immune-regulating cytokines, specifically interleukin-2 (IL-2) and gamma interferon (γ-IFN), in the absence of Concanavalin A (Con A), indicating its potential to activate cell-mediated immune responses. Additionally, it countered the decline in IL-2 and γ-IFN production induced by chromium without affecting the production of interleukin-4 (IL-4), suggesting a specific immunomodulatory effect of SBT (151). *In vivo* studies involving male albino rats further supported SBT’s protective properties. An alcoholic leaf extract of SBT protected animals from oxidative damage induced by chromium exposure (150). The extract also demonstrated the capability to safeguard glial cells against oxidative damage induced by hypoxia (153). Moreover, triterpenoids from SBT displayed inhibitory effects on nitric oxide production and enhanced radical-scavenging activities, indicating their potential as antioxidants (154). Additional research by Kim et al. (152) focused on assessing the antioxidant and α-glucosidase inhibitory activity of SBT leaf extracts. Several compounds were isolated from SBT leaf extracts, and the butanol fraction, which contained the highest phenolic compound content, exhibited potent radical-scavenging activity and significant α-glucosidase inhibitory effects. Furthermore, SBT leaf extract has demonstrated anti-inflammatory properties in various studies. It was found to possess anti-inflammatory activity in adjuvant-induced arthritis (AIA) rat models and to counter lipopolysaccharide-induced inflammatory responses in murine macrophages (155, 157). Casuarinin, isolated from SBT leaves, showed inhibitory effects on TNF-α-induced ICAM-1 expression in human keratinocytes, suggesting its potential as an anti-inflammatory agent (156). In murine macrophage cell lines, SBT leaf alcoholic extract significantly inhibited the enhanced production of nitric oxide induced by lipopolysaccharide (LPS), partly through its inhibitory effect on inducible nitric oxide synthase (iNOS) activation (157). Lastly, recent research has indicated that SBT leaf alcoholic extract can enhance the antigen presentation ability of macrophages in aged mice, suggesting its potential as an immune-boosting and anti-aging agent (364).

Studies have investigated the hepatoprotective activity of SBT leaves and seed oil in animal models with carbon tetrachloride (CCl4)-induced liver damage, yielding promising results. Researchers, including Geetha et al. (158) and Hsu et al. (159), observed that both SBT leaf alcoholic extract and seed oil displayed hepatoprotective effects by mitigating CCl4-induced liver injury. Additionally, in a recent study by Maheshwari et al. (160), the oral administration of phenol-rich fraction PRF significantly protected against CCl4-induced liver damage. This protection was reflected in reduced levels of enzymes like aspartate aminotransferase, alanine aminotransferase, γ-glutamyl transpeptidase, and bilirubin in the serum, along with enhanced hepatic antioxidant activity (161, 162).

The adaptogenic properties of SBT leaf extract have been investigated in rat experiments. The results demonstrated significant anti-stress and adaptogenic effects of the extract (163). Furthermore, the impact of the extract on lipid peroxidation and antioxidant parameters in the liver and gastrocnemius muscle of rats was investigated. The findings indicated that supplementation with SBT leaf extract effectively reduced oxidative stress in both liver and muscle tissues during exposure to C–H–R stress and in the post-stress recovery period (164). Treatment with SBT leaf extract helped maintain tissue glycogen levels and enzyme activities (including hexokinase, phosphofructokinase, citrate synthase, and glucose-6-phosphate dehydrogenase) in the blood, liver, and muscle. This suggests that SBT leaf extract treatment had a positive impact on shifting metabolism from anaerobic to aerobic pathways during multiple stress exposures and post-stress recovery (162).

Flavonoids found in SBT fruit and leaves have garnered attention for their potential to improve cardiovascular health and address various health concerns, with isorhamnetin and quercetin being among the primary components. Treatment with these extracts has shown protective effects against conditions such as myocardial ischemia and reperfusion injury, tumors, oxidative damage, and aging (167). In another study, flavonoids from SBT were found to reduce the production of pathogenic thromboses in mice (166). Additionally, these flavonoids protected endothelial cells from injuries caused by oxidized low-density lipoprotein, a contributing factor in cardiovascular disease, by regulating the expression of LOX-1 and eNOS (165). Furthermore, in research involving high-fat-fed mice, it was observed that SBT leaves (SL) and their flavonoid glycosides (SLG) had beneficial effects. They reduced adiposity by suppressing lipogenesis in adipose tissue while increasing energy expenditure. SL and SLG also improved hepatic steatosis by suppressing hepatic lipogenesis and lipid absorption, while enhancing hepatic fatty acid oxidation. These effects were associated with an improvement in dyslipidemia. Moreover, SL and SLG improved insulin sensitivity by suppressing plasma GIP levels, which are influenced by secreted resistin and pro-inflammatory cytokines, and by modulating hepatic glucogenic enzyme activities. In particular, SLG appeared to offer significant protection against the adverse effects of diet-induced obesity (DIO) and its associated metabolic complications, including adiposity, dyslipidemia, inflammation, hepatic steatosis, and insulin resistance (162, 168). These findings highlight the potential of SBT flavonoids in promoting cardiovascular health and managing metabolic issues related to obesity and insulin resistance.

A systematic chemical investigation of active fractions from SBT leaves has revealed a novel phytochemical compound called Hiporamin. Hiporamin is a purified fraction of polyphenols primarily composed of monomeric hydrolyzable gallo-ellagic-tannins (170). Hiporamin exhibits potent anti-viral activity, particularly against Influenza and *Herpes* viruses. Its ability to inhibit viral neuraminidase is one of the mechanisms through which it exerts its anti-Influenza virus activity. Additionally, it has shown inhibitory effects against HIV infection in cell culture and has displayed antimicrobial activity. Furthermore, SBT leaf extract has exhibited significant anti-dengue activity when evaluated in Dengue virus type-2-infected human macrophages derived from blood. This activity is associated with changes in the levels of TNF-α and IFN-γ (169). Moreover, both aqueous and hydroalcoholic leaf extracts of SBT have demonstrated growth-inhibiting effects against various bacteria, including *Bacillus cereus*, *Pseudomonas aeruginosa*, *Staphylococcus aureus*, and *Enterococcus faecalis* (171).

SBT leaf extracts, whether in aqueous or alcoholic form, have demonstrated impressive radioprotective effects, significantly increasing the survival rate of mice exposed to lethal doses of radiation. Moreover, SBT leaf extract has been effective in mitigating radiation-induced damage to the hemopoietic system and restoring the ferric-reducing activity of plasma (173, 174). A study by Bala et al. (172) further supports the radioprotective properties of SBT leaf extract. It is suggested that the high content of phenolic compounds and thiols in the extract may contribute to radiation protection by neutralizing radiation-induced oxidative stress, supporting stem cell proliferation, and facilitating tissue regeneration.

Recent scientific research has unveiled the remarkable wound-healing properties of SBT leaf extract, particularly in the context of acute and chronic dermal wounds, including burns and diabetic wounds, in rat models. Animals treated with SBT leaf extract experienced a significantly faster reduction in wound area compared to both control and standard care (silver sulfadiazine-treated) animals. The topical application of SBT extract led to increased neovascularization, collagen synthesis, and stabilization at the wound site. This was substantiated by the upregulation of key factors such as VEGF (vascular endothelial growth factor), collagen type-III, and matrix metalloproteinases (MMP-2, MMP-9). Additionally, the levels of hydroxyproline and hexosamine, which are indicative of collagen production and tissue repair, were found to be elevated in the SBT-treated animals (175). Furthermore, SBT treatment increased endogenous enzymatic and non-enzymatic antioxidants while simultaneously reducing lipid peroxide levels in the granulation tissue of the wounds. This antioxidant activity likely plays a critical role in promoting effective wound healing. Importantly, SBT leaf extract is safe, with no cytotoxicity concerns, heavy metal contamination, or adverse effects after oral administration, further emphasizing its potential as a natural and effective wound-healing agent (162).

#### 3.1.6 Strawberry tree (*Arbutus unedo* L.)

Orak et al. (93) investigated the antimicrobial activity of *Arbutus unedo* L. leaves against three bacteria: *Salmonella enteritidis*, *Escherichia coli*, and *Staphylococcus aureus*. They also tested antifungal activity against two aflatoxigenic molds: *Aspergillus parasiticus* NRRL 465, and *Aspergillus parasiticus* NRRL 2999. The leaf extract showed growth inhibition halos for agar well diffusion assay against *Staphylococcus aureus*; meanwhile, it showed no antibacterial activity against *Salmonella enteritidis* and *Escherichia coli*, which are Gram+ bacteria. The reason why Gram+ bacteria are more susceptible than Gram− bacteria is likely due to differences in cell membrane components and their arrangement. In terms of antifungal activity, it was found that the inhibitory effect of the leaf extract against *Aspergillus parasiticus* NRRL 465 was lower than against *Aspergillus parasiticus* NRRL 2999. These results could be helpful and suggest that strawberry tree leaves may be used in pharmaceuticals and the functional food and nutraceutical industries as a source of antioxidants (93).

#### 3.1.7 Figs (*Ficus* spp.)

Salem et al. (92) conducted a systematic study to test different parts of figs for antibacterial activity. Extracts of *Ficus retusa* (bark, wood, leaves) showed moderate activity against some selected bacteria. In particular, methanol extract (MeOH) has shown excellent activity against several bacteria such as *Agrobacterium tumefaciens*, *Serratia marcescens*, *Pseudomonas aeruginosa*, *Escherichia coli*, *Staphylococcus aureus*, *Bacillus subtilis*, and *Bacillus cereus* (92). The ethanolic extract of *Ficus binjamina* leaves inhibited all viruses studied: Var*icella-Zoster Virus* (VZV), and *Herpes Simplex Virus-1* and *-2* (HSV-1 and HSV-2). In a study by Jeong et al. (176), the antibacterial activity of MeOH extract showed strong activity against *Porphyromonas gingivalis*, *Aggregatibacter actinomycetemcomitans*, *Prevotella intermedia*, *Streptococcus anginosus*, and *Streptococcus gordonii*. Among various *Ficus tsiela* leaf extracts, diethyl ether extract showed the best inhibitory effect on *Klebsiella pneumoniae*, *Escherichia coli*, and *Pseudomonas aeruginosa*. Finally, a decrease in activity against *Staphylococcus aureus* was observed (177).

#### 3.1.8 Pomegranate (*Punica granatum*)

Pomegranates have represented fertility and prosperity throughout history. Additionally, different pomegranate parts have been employed in traditional medicine to treat a range of ailments. Pomegranate fruits are reputed to be used for expelling parasites, the seeds and fruit peels as a remedy for diarrhea, the flowers to manage diabetes, the tree bark and roots to stop bleeding and heal ulcers, and the leaves to regulate inflammation and address digestive system problems (280, 281). The bioactivities of polyphenols in pomegranate fruits, particularly anthocyanins and hydrolyzable tannins (HTs), have received the majority of research attention thus far, although the pomegranate produces and accumulates a wide variety of phytochemicals with different structures in diverse tissues. The HTs in pomegranate leaves are substantially distinct from those in the fruit peel. Leaves primarily contain granatins A and B, while punicalins and punicalagin are present in minuscule amounts (365). Moreover, derivatives of ellagic acid and ellagitannins, such as brevifolin, brevifolin carboxylic acid, and urolithin M-5, have been extracted from pomegranate leaves (366). Similarly to other plants, pomegranate leaves also contain high levels of flavone glycosides (e.g., luteolin and apigenin) (366). N-(20,50-dihydroxyphenyl)pyridinium chloride was discovered in pomegranate leaves in addition to the alkaloids that accumulate in stem barks and roots (366).

### 3.2 Tropical fruits

#### 3.2.1 Guava (*Psidium guajava*)

Guava, scientifically known as *Psidium guajava* (L.), is a widely cultivated fruit that is part of the *Myrtaceae* family. It is mainly grown in tropical regions, such as South-Central Asia, Indonesia, and Central and South America (178, 367). Treatment usually includes decoctions of the bark, roots, and leaves (284). The main known traditional use is in Latin America and the Caribbean to treat diarrhea and stomach pain due to indigestion (285–287). Other common uses include the treatment of gastroenteritis, dysentery, and colic due to their antibacterial activity against pathogens of the intestine. Its medicinal use has been well documented among Indigenous groups of the Tikuna Indians, Mexican Indians, Nahuatl, Maya, Popoluca, and Zapotec. The leaves are commonly chewed to relieve toothache and applied to ulcers, rheumatic pains, and wounds (282, 289). Waterleaf extract is used to lower blood sugar levels in diabetics (283), and for the control, management, and/or treatment of other various human ailments, including hypertension, in South Africa, where the leaves of *Psidium guajava* have traditionally been used in folk medicine (288). It is also used in Venezuela as an astringent. In Uruguay, a decoction of the leaves is used as a uterine and vaginal cleanser, especially for leucorrhea (284). The shoots and leaves are used by the West Indians for antispasmodic and antipyretic baths. Leaf powder is used to treat cholera and epilepsy, and guava leaf tincture is given to children suffering from seizures (276).

Many studies have shown that the health benefits of guava leaves are due to bioactive compounds such as flavonoids, polysaccharides, and phenols, which have a variety of biological effects, including antioxidant, antihypertensive, antidiabetic, antidiarrheals, anticancer, antimicrobial, lipid-lowering, and hepatoprotective effects (178, 179). Additionally, extracts from *P. guajava* leaves have been investigated for their potential in treating a range of diseases caused by fungi, viruses, bacteria, and protozoa (e.g., AIDS, rotavirus disease, influenza, herpes, cholera, gastrointestinal and mucocutaneous infections, stomach ulcers and gastritis, urinary infections and venereal diseases, periodontal and oral infections, giardiasis, malaria, trichomoniasis, amoebiasis, and leishmaniasis) (181).

Extracts from guava leaves have been studied to be used in bioactive films, blended with sodium alginate and at different proportions of ethanol and water extracts, to enhance the antioxidant and antibacterial properties in food packaging materials. As revealed by HPLC-PDA analysis, the main phenolic compounds in the ethanol extract were quercetin, isoquercitrin, kaempferol, rutin, avicularin, quercetin-3-O-β-D-xylopyranoside, and quercitrin; while in the water extract were quercetin, ellagic acid, avicularin, quercetin-3-O-β-D-xylopyranoside, and gallic acid. Results indicate that the incorporation of guava leaf extract and sodium alginate into food packaging materials increases their antimicrobial and antiradical potential (180).

The hydrodistillation of *Psidium guajava* leaves and the subsequent GC–MS analyses of the resulting essential oil revealed a total of 53 compounds, with (E)-nerolidol the most abundant, followed by (E)-caryophyllene, (2Z,6E)-farnesol, and ledol. Although the oil showed no antimicrobial or cytotoxic effects, it did display notable larvicidal activity against *Chaoborus plumicornis* as well as marginal nematicidal (*Caenorhabditis elegans*) and insecticidal (*Drosophila melanogaster*) activities (84).

#### 3.2.2 Loquat (*Eriobotrya japonica* L.)

Loquat (*Eriobotrya japonica* L.), belonging to the *Rosaceae* family, is a semitropical fruit tree widely distributed in Southeastern China. Its leaves are used as a famous traditional Chinese medicine and a popular tea material (290, 368), and have been used to treat various diseases like inflammation, cough, pain, asthma, chronic bronchitis, diabetes (290).

Research on anti-inflammatory agents frequently uses an experimental model of inflammation brought on by lipopolysaccharide (LPS). In rats with LPS-induced chronic bronchitis, loquat leaf extracts high in triterpene acids, particularly ursolic acid, had anti-inflammatory effects on alveolar macrophages (186). Twelve triterpene acids, including one of the lupane type, four of the oleanane type, and seven of the ursane type, were found to have significant anti-inflammatory effects when used to treat mice with 12-O-tetradecanoylphorbol-13-acetate (TPA)-induced ear edema (182). Loquat leaf extract and its triterpene ursolic acid suppressed the LPS-induced cytokines and the inducible enzyme production via the NF-κB signaling pathway in A-549 cells, which are human lung epithelial cells (185). Loquat leaf extracts suppressed the expression of a wide range of inflammation-related genes in LPS-stimulated human gingival fibroblasts (183). Another potential additional chemical mechanism for its anti-inflammatory benefits is antioxidant activity (369). The level of cellular oxidative stress affects NF-κB activation, and antioxidants as methyl chlorogenic acid derived from loquat leaves can prevent redox-sensitive NF-κB activation and suppress NF-κB-dependent gene expression (184).

Loquat leaf among 56 chosen Chinese medicinal herbs had stronger antioxidant capabilities than 54 other medicinal plants (187). Quercetin-3-rhamnoside, kaempferol-3-rhamnoside, methyl chlorogenate, quercetin-3-sambubioside, and chlorogenic acid isolated from loquat leaf extract all demonstrated notable inhibitory activity against free radical generation (370). In another study, arbutin, quercetin-3-O-l-rhamnoside, epicatechin, cinchonain Ia and Ib have been identified as the significant antioxidants in loquat leaf that demonstrate strong antioxidant activity (371, 372). Different loquat fruit and leaf extracts demonstrated protective properties against intracellular ROS utilizing various cell models (152, 188, 189, 373). When the antibiotic chloramphenicol was applied to leukocytes and erythrocytes, loquat fruit extract dramatically reduced the production of ROS and NO (373). In HepG2 cells, ethanol extracts from loquat leaves showed hepatoprotective benefits against ethanol-induced toxicity. Intracellular ROS production was reduced, hepatic antioxidant activity increased, and cellular survival was also raised (188). The antioxidant enzyme activity of SOD, catalase, glutathione-S-transferase, glutathione peroxidase, glutathione reductase, and reduced glutathione were all considerably boosted by loquat leaf extract in HepG2 cells (188). Treatment with loquat leaf effectively reduced the production of intracellular ROS by the A1-42 peptide and prevented the death of neuronal cells in another cell model employing amyloid-induced oxidative stress in neuronal PC12 cells (189).

Recent studies have demonstrated the effectiveness of loquat leaf or seed extracts in the prevention and management of type-1 and type-2 diabetes (186). In alloxan-diabetic rats, an ethanol extract of loquat leaves demonstrated a considerable hypoglycemic impact by reducing blood glucose levels (190). Nerolidol-3-O-l-rhamnopyranosyl, a sesquiterpene glycoside, and euscaphic acid, a triterpene acid, dramatically reduced plasma glucose levels in alloxan-diabetic mice, indicating that they are major active hypoglycemic compounds in loquat leaf (374, 375). In a hypercholesterolemic zebrafish model fed a high-cholesterol diet, loquat leaf extracts significantly reduced the rise in blood glucose, total cholesterol, and triglyceride levels (191). Qadan et al. (376) discovered that cinchonain Ib increased insulin secretion from INS-1 cells and may have an insulinotropic effect for treating type 2 diabetes. In 3 T3-L1 adipocytes, corosolic acid, which was extracted from loquat leaf, increased the absorption of 3H-glucose, prevented the development of preadipocytes into adipocytes, and downregulated the expression of PPAR and the CCAAT/enhancer binding protein. Corosolic acid may therefore control glucose metabolism without increasing body fat (377).

Both loquat leaf extracts made from water and ethanol prevented mice from developing breast cancer after being exposed to the carcinogen 7,12-dimethylbenz[a]anthracene (DMBA) (189). Both extracts dramatically reduced the beginning and proliferation of tumor cells, which prevented the spread of breast cancer. Numerous investigations have shown that loquat extract is cytotoxic to many cancer cell types. Loquat leaf showed high cytotoxicity in cell lines of the lung (A549) carcinoma, cervical epithelioid (HeLa), and estrogen receptor-negative breast cancer (MDA-MB-231) during an assessment of 14 eastern medicinal herbs for antiproliferative activity (192). Roseoside derived from loquat leaf was discovered to be the primary chemical that greatly slowed down carcinogenesis generated by peroxynitrite as an initiator and TPA as a promoter in a two-stage carcinogenesis experiment on mouse skin (193). Euscaphic acid showed strong antitumor-boosting effects on mice tumors generated by 7,12-DMBA as an initiator and TPA as a promoter in another two-stage *in vivo* carcinogenesis test (182). The migration and invasion of B16F10 melanoma cells and MDA-MB-231 human breast cancer cells were also significantly inhibited by loquat seed and leaf extracts, which was partially due to the inhibition of matrix metalloproteinase-2 (MMP-2) and MMP-9 (194, 195).

#### 3.2.3 Mango (*Mangifera indica* L.)

Aside from its highly well-known fruits, the mango tree is an evergreen that has a variety of traditional medicinal uses. Pruning produces significant amounts of agricultural leftovers, including leaves, flowers, stems, and bark, in addition to its economically significant portion (fruit), which presents farmers with disposal challenges. Mango leaves (MLs) are typically burned or thrown away because they are considered agricultural waste. However, due to their health benefits, MLs are used in traditional folk tea recipes as well as in the treatment of respiratory and diabetic conditions throughout Asia and Africa (291, 292).

The essential oil from MLs (MLO) is typically extracted using the hydro distillation process, and its chemical composition was examined using gas chromatography and mass spectrophotometer. Monoterpenes, sesquiterpenes, their counterparts, and non-terpenoid and oxygenated hydrocarbons were detected in MLO. Seven chemical components were identified by the MLO profile, including α-humulene, β-elemene, α-gurjunene, camphene, α-copaene, α-pinene, and pinene (378). Sesquiterpenes and the main constituents β-caryophyllene, β-selinene, α-gurjunene, and δ-3-carene were abundant in MLO extracted using the hydro-distillation method (379). Minerals such as manganese, zinc, boron, magnesium, sodium, iron, calcium, nitrogen, phosphorus, and potassium, and vitamins such as C, E, B, and A, are all potentially present in MLs (380). These minerals are essential for human nutrition because they are involved in numerous processes that keep teeth and bones strong, energy metabolism, blood pressure regulation, blood clotting, immune system health, muscle relaxation and contraction, and nerve function, and are a component of numerous enzymes (381). In terms of pharmacological and biological activities, the bioactive phytochemicals found in MLs extracts have a high potential, including anti-tumor, antipyretic, antidiarrheal, antiparasitic, antifungal, anti-obesity, antiallergic, immunomodulatory, antiviral, antimicrobial, anti-inflammatory, antidiabetic, and antioxidant activities (196, 197). Amino acids, fatty acids, vitamins, carotenoids, sterols, polysaccharides, terpenoids, and polyphenols are the main categories of phytochemicals found in MLs. Among them, total phenolic compounds (TPC), including flavonoids, terpenoids, tannins, benzophenones, xanthones, and phenolic acids are most abundant in ML. The effectiveness of TPC against chronic diseases such as neurological, cardiovascular diseases, diabetes, and cancer has been demonstrated by numerous epidemiological studies (382). To combat chronic diseases, TPC regulates a variety of physiological processes including cellular redox potential, signal transduction pathways, cell proliferation, and enzyme activity (383). Mangiferin, a naturally occurring xanthonoid polyphenol antioxidant, is one phenolic bioactive substance in ML, identified by Pan et al. (384) as a significant component of the ML extract, with isoswertisin, quercetin-3-O-D-galactoside, and quercetin-3-O-D-glucoside. Mangiferin has been shown to modulate the expression of inducible nitric oxide synthase and TNFα, improve insulin sensitivity, and reduce cholesterol production (385). In another study, 83 substances were found in ML essential oils of five mango cultivars (386) using gas chromatography–mass spectrometry (GC–MS). The greater amounts of these were α-humulene, α-selinene, and trans-caryophyllene. By using a nuclear magnetic resonance (NMR) spectroscopic method, Gu et al. (387) identified and characterized four benzophenone compounds, norathyriol, mangiferin, manindicin A, and manindicin B, from ML extract.

Due to their antioxidant and anti-inflammatory properties, polyphenols found in MLs like quercetin, mangiferin, phenolic acids, and gallotannins have been shown to have chemo-preventive benefits against different cancer types (388). The principal bioactive xanthone glucoside, mangiferin, is primarily responsible for the antitumoral properties of the MLs extract. These substances have been shown to inhibit the proliferation, migration, and invasion of numerous malignancies (389). Mangiferin’s capacity to control the expression of metalloproteinases, which controls cell proliferation and prevents the epithelial-mesenchymal transition, which ultimately results in a lack of cell adhesion, may also be responsible for its antimetastatic and anti-invasive properties.

Due to their anti-diabetic pharmacological compounds like flavonoids (quercetin and its glucoside derivatives) and benzophenones (mangiferin), MLs have been widely touted as useful ethnomedicine against diabetes mellitus (DM). Inhibiting the α-glucosidase and α-amylase enzymes, which control postprandial glucose absorption, is one of the most successful methods for treating DM (390). Mangiferin and MLs extracts were compared to see which extract was more effective at inhibiting α-glucosidase enzymes. The management of diabetes and the suppression of α-glucosidase enzyme activity both seem to include the active compound mangiferin (391). Four bioactive chemicals were extracted and identified as manindicins A and B, mangiferin, and norathyriol (deglycosylated mangiferin) from MLs extract (387). According to the authors, norathyriol showed strong α-glucosidase inhibition, which was four times as effective as the commercial inhibitor acarbose. Manindicin A, manindicin B, and mangiferin showed weaker α-glucosidase inhibition. Mangiferin’s molecular size and polarity may be the cause of its lower inhibitory potential.

Mango leaves contain a variety of compounds that have antibacterial properties, including tannins, terpenes, glycosides, saponins, alkaloids, and phenolics. Shikimic acid, ellagic acid, ethyl digallate, kainic acid, quercetin, catechin, hyperin, gallic acid, and protocatechuic acid are among the polyphenols and phenolic acids found in ML extract that have antimicrobial properties (392). Mangiferin has also demonstrated high iron chelating activity, which favors antibacterial action. A myriad of terpenes identified from MLs extract including α-farnesene, α-terpinolene, α-guaiene, α-pinene, β-pinene, β-ocimene, β-elemene, δ-elemene, γ-cadinene, γ-terpinene, limonene, myrcene, humulene, linalool, friedelin, lupeol, camphene, and taraxerol exhibit bactericidal and bacteriostatic effects against different pathogens (392). The gallatotannins were identified in the leaf extract by HPLC-TOF-ESI/MS analysis. Gallatotannins’ antimicrobial activities have been linked to their capacity to encourage the chelation of metal ions, disrupt lipid bilayer membranes, and block the pathogen’s enzymes (393). Bharti (394) noted that *Enterobacter aerogenes* and *Mycobacterium tuberculosis* are both successfully inhibited by hexane/ethyl acetate and hexane extracts of MLs. Ouf et al. (386) demonstrated that the essential oils extracted from the leaves of five Egyptian mango cultivars could be used as preservative materials against *Salmonella typhi*, *Aspergillus flavus*, *Pseudomonas aeruginosa*, *Escherichia coli*, *Bacillus subtilis*, and *Staphylococcus* spp.

To assess the hypocholesterolemic activity of the methanolic extract of the MLs, the *in-vitro* pancreatic cholesterol esterase inhibition assay was used, and 3b-taraxerol exhibited hypocholesterol activity (82). In a separate investigation, the anti-obesity effects of MLs extracts from the Ubá variety were assessed in obese rats (male Wistar rats) fed a high-fat diet (395). The authors concluded that MLs can treat obesity by regulating the expression of transcriptional factors and adipogenic enzymes.

Mango aqueous young leaf extract has been shown to have inhibitory effects on Gram-negative microorganisms involved in gastrointestinal illnesses, according to De et al. (396). The phytochemicals in the crude extract, according to the authors, are a key component of the antidiarrheal activity. Aqueous extract of MLs was tested against many pathogens, including *S. sonnei*, *S. typhi*, *E. coli*, and *Vibrio cholera*. It was found that as the dose level increased, the antidiarrheal activity also did so. Thus, it was determined that the mango aqueous young leaf extract can be used to treat cases of diarrhea.

#### 3.2.4 Papaya (*Carica papaya*)

Papaya leaves have been shown to contain many active constituents such as glucosinolates, cyanogenic glucosides, flavonoids, ascorbic acid, α-tocopherol, cystatin, chymopapain, and papain (397). In these leaves, compounds with potential antitumor activity include α-tocopherol, lycopene, flavonoid, and benzyl-isothiocyanate (98). Otsuki et al. (98) have examined the potential role of *Carica papaya* (CP) as an anti-cancer therapy, using the aqueous extract of its leaves against various cancer cell lines: a pancreatic adenocarcinoma cell line (Capan1), a mesothelioma cell line (JMN), a breast adenocarcinoma cell line (MCF-7), an anaplastic large cell lymphoma cell line (Karpas-299), a plasma cell leukemia cell line (ARH77), mesothelioma cell lines (MESO-4, H226, and H2452), a pancreatic epithelioid carcinoma cell line (Panc-1), a lung adenocarcinoma cell line (PC14), hepatocellular carcinoma cell lines (HepG2 and Huh-7), a cervical carcinoma cell line (HeLa), a chronic myelogenous leukemia cell line (K562), Burkitt’s lymphoma cell lines (Ramos and Raji), and T cell lines (HPB-ALL, CCRF-CEM, Molt-4, Jurkat, and H9). CP extract has been demonstrated to inhibit the proliferation of mesothelioma (H2452), pancreatic epithelioid carcinoma (Panc-1), lung adenocarcinoma (PC14), hepatocellular carcinoma (HepG2), breast adenocarcinoma (MCF-7), and cervical cancer (HeLa) cells in a dose-dependent way. In addition, CP extract inhibits the proliferative response of hematopoietic cell lines such as anaplastic large cell lymphoma (Karpas-299), Burkitt’s lymphoma (Raji), plasma cell leukemia (ARH77), and T-cell lymphoma (Jurkat). These data suggest that one of the mechanisms involved in the inhibition of tumor cell growth correlates with the induction of cell death, including apoptosis. Apoptosis, or programmed cell death, is a normal and fundamental process that occurs in a highly regulated and precise manner. It plays an important role in the normal development and maturation of tissues and maintains homeostasis in the body by regulating the immune system. Apoptosis is the most powerful defense system against cancer, and many chemopreventive agents act by inducing apoptosis in transformed cells (99). Otsuki et al. (98) found that CP extract reduced IL-4 and IL-2, whereas the production of the anti-tumor-related cytokines, such as TNF-α, IFN-γ, IL-12p70, and IL-12p40 was enhanced without growth inhibition. Thus, CP extracts can alter not only the activation of antigen-presenting cells (APCs) but also the interaction of T-B or T-APC cells. In addition, IFN-γ, TNF-α, and IL-12 are potent factors inducing cell-mediated cytotoxicity, thus there might be a specific relationship between increased production of Th1-type cytokines and increased cytotoxicity, which can lead to enhanced antitumor immunity. This study showed that CP extract had several biological effects *in vitro*. It has an antiproliferative effect on tumor cells, upregulates antitumor genes in human peripheral blood mononuclear cells (PBMC), increases cytotoxicity against tumor cells, and promotes the production of Th1-type cytokines (98).

### 3.3 Citrus fruits (*Citrus* spp.)

One of the most widely cultivated fruit plant species in the world is the citrus fruit (*Rutaceae* Juss. family). Oranges are the fruit that is grown, consumed, and processed the most, followed by grapefruits, mandarins, clementines, tangerines, limes, and lemons. Almost 40–50% of citrus fruit production is intended for industrial processing, so the citrus business generates a lot of waste. Nevertheless, only peel, seeds, and pulp were primarily examined for their richness in advantageous phytochemicals (398). Recently, few studies have been emphasized the value of leaf wastes of smaller citrus species.

#### 3.3.1 Pomelo (*Citrus grandis, Citrus maxima*)

The leaves that are thrown away after pruning the pomelo (*Citrus grandis* (L.) Osbeck “Mato Peiyu”) known as “Mato Peiyu” are considered a waste product in agriculture. Their anti-inflammatory, antibacterial, antityrosinase, and antioxidant properties were assessed to determine whether or not they might be used in skin care products. Citronellal and citronellol were found to be the primary components of *C. grandis* (L.) Osbeck essential oils. The essential oils’ antibacterial activity was demonstrated against *Staphylococcus aureus*, *Candida albicans*, *Escherichia coli*, and *Pseudomonas aeruginosa* (198). Additionally, *C. maxima* leaves, particularly in methanol extracts, have demonstrated positive effects in regulating blood glucose levels and reducing lipid peroxidation (199).

#### 3.3.2 Kaffir lime (*Citrus hystrix*)

*Citrus hystrix*, a citrus species from the Pummelo cluster that is also known as kaffir lime, has historically been utilized as food and medicine, primarily in the Asian region. More recently, however, its leaves have been examined and classified for the presence of significant phytochemicals. The traditional applications of this plant and its potential for use in cosmetics and pharmaceuticals, particularly in the treatment of bacterial and fungal infections, are explained by the chemical structures of the bioactive molecules and the presence of essential oils in the leaves. Terpinen-4-ol, a-citronellol, and citronellal make up the majority of the essential oils of *C. hystrix* leaves, but other minor compounds were also found (citronellol, geranylacetate, p-cimene, apinene, citronellyl acetate, limonene, 1,8-cineole, and alpha-terpineol, geranylacetate, elemol, L-linalool, and delta-cadinene). These essential oils, containing citronellal as the main compound, proved to be the most effective against bacterial strains, while those with mainly terpinene-4-ol lacked antibacterial activity. Moreover, many flavones, flavanones, and flavonoids were isolated from leaves, such as didymin, hesperidin, myricetin, peonidin, cyanidin, quercetin, luteolin, hesperetin, apigenin, and isorhamnetin, as well as rutin and diosmin. Additionally, l,2-di-O-a-linolenoyl-3-O-betagalactopyranosyl-sn-glycerol (DLGG), glyceroglycolipids, and a mixture of l-O-a-linolenoyl-2-O-palmitoyl-3-O-betagalactopyranosyl-sn-glycerol and its counterpart (LPGG) were identified in *C. hystrix* leaves. Different extracts of kaffir lime leaves have shown promising anti-inflammatory, hepatoprotective, and antioxidant properties, even in the presence of different phenolic compounds. Furthermore, a methanolic extract of *C. hystrix* fresh leaves exhibited an anti-tumor effect due to the presence of DLGG. Essential oils extracted from the fruit and leaves of *C. hystrix* were also found to possess anti-tumoral activity against mouse leukemia cells (P388 cell) and cervical cancer cells (KB cell), with the ethyl acetate fraction from leaves being the most cytotoxic, inducing the highest apoptosis rate in HeLa cells. The research findings indicated that the extract, especially the chloroform extracts, had a noteworthy cytotoxic impact on cervical cancer (HeLa) and neuroblastoma (UKF-NB3, IMR-5, SK-N-AS parental) cell lines, as demonstrated through cell viability assessments. Essential oil from *C. hystrix* fresh leaves also demonstrated tyrosinase inhibitory activity, as well as antifertility, cardioprotective, and hepatoprotective, effects. Moreover, an ethanolic extract of keffir lime leaves displayed larvicidal effects against *Aedes aegypti* (200, 201). The leaves of the kaffir lime calamansi (*Citrus microcarpa*) are characterized by their rich chemical composition, which includes a wide range of compounds, including monoterpenes, monoterpene alcohols, sesquiterpenes, aromatics, aldehydes, hydrocarbons, and esters. Remarkably, these leaves surpass the peels in terms of chemical diversity. The main chemical constituents of calamansi leaves include citronella, citronellol, 3-carene, and 𝛽-phellandrene. Furthermore, the essential oil extracted from calamansi leaves shows a strong inhibitory effect against bacteria such as *Staphylococcus aureus*, *Pseudomonas aeruginosa*, *Escherichia coli*, and *Streptococcus mutans*, suggesting promising antimicrobial properties (202).

#### 3.3.3 Karakaya citron (*Citrus medica*)

*Citrus medica*, including its Salib fruits and leaves, has a rich history of traditional use for addressing various health concerns (399). Studies have revealed diverse bioactive properties associated with different parts of the plant. The leaves are rich in phenols and flavonoids, including rutin, quercetin, and hesperidin, which have been associated with various health benefits (204). For instance, *C. medica* leaves have shown cytotoxic effects on earthworms, suggesting bioactivity (203). Moreover, *Citrus medica* cv. Diamante leaves have exhibited significant hypoglycemic potential by inhibiting α-amylase, an enzyme involved in carbohydrate digestion (204). Additionally, *Citrus medica* L. leaves have shown estrogenic activity, making them a subject of interest for further research (205).

#### 3.3.4 Lime (*Citrus aurantifolia*)

*Citrus aurantifolia* (lime) is extensively utilized by the food industry for candy and liqueur production. It delves into the plant’s chemical diversity, antioxidant properties, and potential health benefits, which can vary due to factors like soil composition, solar exposure, and geographical location. The findings suggest that both the peel and leaves of *Citrus aurantifolia* are promising sources of bioactive compounds for supplements or nutraceutical products. Particularly noteworthy is the presence of flavonoids such as rutin, apigenin, quercetin, kaempferol, nobiletin, tangeretin, and hesperidin, known for their antioxidant and anti-inflammatory properties, potentially beneficial in addressing conditions like Alzheimer’s disease (206). Additionally, *Citrus aurantifolia* leaves have demonstrated *in vitro* antibacterial effects against a range of bacteria, including Gram-positive (e.g., *Bacillus subtilis*) and Gram-negative (e.g., *Salmonella*, *Escherichia coli*) strains (207).

#### 3.3.5 Bitter orange (*Citrus aurantium*)

*Citrus aurantium* leaves are abundant in bioactive compounds, notably flavonoids and phenolic constituents. These leaves have applications in the pharmaceutical industry and contain essential oils like limonene and linalool. Numerous studies have identified the presence of flavonoids, phytosterols, tannins, terpenoids, and proteins in *Citrus aurantium* leaves. Extracts from *Citrus aurantium* leaves, including those prepared with water, ethanol, and chloroform, have shown antibacterial activity against both gram-positive (*Staphylococcus aureus* and *Bacillus subtilis*) and gram-negative bacteria (*Escherichia coli* and *Klebsiella pneumonia*) (208).

#### 3.3.6 Sweet orange (*Citrus sinensis*)

Acetone and hexane extracts of *C. sinensis* (sweet orange) exhibited antibacterial activity against *Helicobacter pylori* (209). Orange leaf extracts induced inotropic depression in guinea pig atria, and these effects were not influenced by common cardiovascular drugs (210). Ethanol extracts from orange leaves and peel increased bone mineral content and density, potentially aiding in osteoporosis management (211). Orange leaf essential oils displayed insecticidal activity against mosquito larvae, offering protection against mosquito bites. Key compounds such as terpineol and 1,8-cineole were effective against mosquito bites (212). Orange leaf extracts exhibited strong bioactivity against various pests, making them a potential natural alternative for crop protection (213). In diabetic rats, intraperitoneal administration of *C. sinensis* leaf essential oil has led to improved glucose control and liver glycogen levels (214).

#### 3.3.7 Mandarin (*Citrus reticulata*)

Leaves from the Murcott mandarin, a hybrid of *C. reticulata* and *C. sinensis*, have been found to protect against alcohol-induced gastric ulcers in rats. These effects are attributed to their anxiolytic properties, along with their ability to reduce inflammation, act as antioxidants, and prevent cell apoptosis (215). *Citrus reticulata* Blanco, or mandarin, leaves and fruit peels have displayed anti-inflammatory characteristics by reducing markers of inflammation in cell cultures (216). By-products of clementines, including their peels and leaves, are rich in antioxidants and may have the potential to serve as functional foods that can help prevent diseases associated with oxidative stress, such as type 2 diabetes and obesity (217).

#### 3.3.8 Grapefruit (*Citrus paradisi*)

*Citrus paradisi*, or grapefruit, leaves, and peel oil contain components like limonene and β-phellandrene, which have been shown to reduce inflammation and the size of edema in rats, indicating their anti-inflammatory properties (218).

#### 3.3.9 Lemon (*Citrus limon*)

*Citrus limon*, or lemon, leaves, known for their content of sabinene, 3-carene, limonene, β-ocimene, citronellal, and citronellol, possess antibacterial properties against both Gram-negative (e.g., *Salmonella typhimurium*, *Pseudomonas aeruginosa*) and Gram-positive (e.g., *Staphylococcus*) bacteria, and they also have potential as insecticides (207, 219).

### 3.4 Pome fruits

#### 3.4.1 Apple (*Malus domestica* Borkh.)

Apple (*Malus domestica* Borkh.) is a fruit that is widely enjoyed for its flavor, aroma, and health benefits. A large portion of this production is either eaten fresh or converted into value-added products like processed juice or cider (400). In recent years, the apple leaf has also been studied for its concentration of bioactive compounds. Rana et al. (301) analyzed *Malus domestica* leaves, collected from the orchard in the mid-hills of the North-western Himalayas. The apple leaf extracts studied revealed the presence of phloridzin, quercetin-3-O-glucoside, phloretin, quercitrin, and epicatechin, as bioactive compounds showing an appreciable free radical scavenging activity (301). In particular, quercetin glycosides are present in high concentrations in apple leaves, including quercetin-3-O-rutinoside, a compound not typically found in apple fruits. Quercitrin, among quercetin glycosides, is identified as a major compound in ethanol extracts of apple leaves (401). While quercetin derivatives are not the primary polyphenolic components of apples, they hold significant importance for human health. Quercetin has been shown to inhibit the growth of human prostate and lung cancer cells and reduce the risk of cardiovascular diseases (402).

Notably, these leaves have higher levels of essential amino acids compared to fruits, which is important as these amino acids are essential for human health (402). Leaves are also excellent sources of essential minerals, particularly calcium, magnesium, iron, and potassium. They also have high levels of organic acids and lower sugar content compared to fruits (402). Apple leaves are particularly rich in flavonol glycosides and dihydrochalcones, unique to apple leaves and fruits (402). Recent research has revealed a wide range of biological activities associated with these leaves, including antioxidative, antibacterial, anti-inflammatory (inhibiting COX-1 and COX-2 activity), anticancer (against prostate cancer cells), and immunosuppressive potentials (37). Leaves exhibit a more diverse composition of anthocyanins compared to fruits, with concentrations up to tenfold higher in leaves. These compounds have strong UV-absorbing properties and accumulate mainly in epidermal cells (403). Regarding carotenoids, both fruits and leaves are rich in 9-cis or 9-cis’-lutein, but levels vary by cultivar. Leaves contain up to four times more carotenoids than fruits. Chlorophyll levels are considerably higher than carotenoid levels, especially in leaves, where the chlorophyll fraction includes chlorophylls and pheophytin as a/b and a’/b’ (402). Characteristic compounds like ursolic and oleanolic acids are found in apple leaves. Although there is no significant difference in the total content of triterpenes between leaves and fruits, leaves have significantly higher levels of specific triterpenes such as uvaol, α-boswellic, betulin, betulinic, maslinic, and tormentic acids (402). Furthermore, these leaves exhibit notable inhibitory effects on enzymes such as COX-1, COX-2, α-amylase, α-glucosidase, pancreatic lipase, AChE, BuChE, and 15-LOX, particularly due to the presence of bioactive compounds (37).

#### 3.4.2 Wild apple (*Malus pumila*)

Wild apples (*Malus pumila*) leaves are considered an alternative source of antioxidant polyphenols. In a recent study, the antioxidant polyphenols from *Malus pumila* leaves were isolated and identified to further evaluate their *in vitro* antioxidant activity. The main isolated polyphenols were Isoquercitrin (IQ), Phloridzin (P), Quercetin-3-O-rhamnoside (QR), Quercetin-3-O-arabinoside (QA), and Quercetin-3-O-xyloside (QX). All five polyphenols have shown significant antioxidant activities, especially IQ (223).

#### 3.4.3 Quince (*Cydonia oblonga*)

The traditional medicinal used various parts of the quince plant, attributing their therapeutic potential to secondary metabolites like tannins, terpenoids, and alkaloids (404–408). Quince leaves are commonly used in decoctions to alleviate nervousness, dysuria, insomnia, cough, cold, abdominal cramps, diarrhea, fever, and hyperglycemia (293–296).

Quince leaves possess protective effects on spermatogenesis in hypercholesterolemia, anti-fungal properties, renoprotective potential, anti-atherogenic and hepatoprotective effects, anti-proliferative effects against colon cancer cells, and antioxidant potential due to the presence of valuable bioactive (224–229). Chemical analysis of Portuguese quince leaves has revealed the presence of compounds like 5-O-caffeoylquinic acid, 3,5-O-dicaffeoylquinic acid, quercetin-3-O-rutinoside, kaempferol-3-O-rutinoside, quercetin-3-O-galactoside, kaempferol-3-O-glycoside, and kaempferol-3-O-glucoside (409). Additionally, flavonoids such as rutin were identified as the most abundant in Tunisian quince leaves (410). Phenolic compounds like chlorogenic acid and organic acids such as D-(−)-quinic acid, oxalic acid, malic acid, and citric acid were also found in quince leaves, which contribute to their antioxidant properties (229, 411). Moreover, essential oils extracted from quince leaves during different seasons contained various compounds such as aldehydes, fatty acids, monoterpenes, norisoprenoids, sesquiterpene hydrocarbons, and benzaldehyde (412, 413).

### 3.5 Stone fruits

#### 3.5.1 Neem (*Azadirachta indica*)

The parts of *Azadirachta indica*, such as fruits, seeds, leaves, roots, and bark have been utilized since ancient times for multiple purposes, including treatment of human ailments (e.g., malaria) and pest control due to its insecticidal properties (220). This is because neem has great versatility, both in its range of beneficial applications and the variety of constituents it contains that are responsible for its different effects (220). Leaves have been reported to be effective for anorexia and skin problems. Anti-inflammatory and antipyretic properties have been attributed to compounds from the leaves, seeds, and oil (230, 231). The most abundant compound in *Azadirachta indica* is the tetranortriterpenoid azadirachtin (AZA), found in higher amounts in seeds than in the leaves (414). Its potential as an insecticide has already been tested and proven (220). It has been shown to have antifungal activity against *Penicillium expansum* (221) and activity in suppressing *Trichophyton* protease (415), aflatoxin formation of *A. parasiticus* (416), and antifeedant activity (222). *In vitro*, the leaves also prevented the development of *M. tuberculosis*, *Klebsiella pneumoniae*, *Vibrio cholerae*, and *M. pyogenes* (417). The presence of various compounds other than AZA may be the cause of the antibacterial action against *S. aureus*, which suggests that the leaf extract can be employed to combat this significant pathogenic microbe.

#### 3.5.2 Blackthorn (*Prunus spinosa*)

According to Shi et al. (418), the genus *Prunus* has many economically significant species that yield edible fruits such as almonds, apricots, cherries, peaches, and plums. However, it also includes certain species that grow naturally, such as *Prunus spinosa* (blackthorn). Blackthorn, often known as “sloe,” is a perennial shrub found across the Northern Hemisphere. It can be found growing along highways and channels, on slopes of large, uncultivated areas, and as windbreaks (419). Blackthorn is well-known in folk medicine as an anti-septic, anti-inflammatory (232), digestive, diuretic, purgative, and astringent agent (297, 298), as well as a mild laxative (299).

The identification and quantification of four anthocyanins (pelargonidin, malvidin, cyanidin, and delphinidin) from leaf extracts of *P. spinosa* was performed by HPLC analysis. In *P. spinosa* leaves, quercetin glycosides and phenolic acids were found by Varga et al. (420). In blackthorn leaves were also identified kaempferol and quercetin glycosides, and phenolic acids by Owczarek et al. (421).

The relationships between the quantities of anthocyanins, flavonoids, and phenols and the results for FRAP, ABTS, and DPPH indicate that polyphenols, particularly anthocyanins, are likely substantial contributions to the antioxidant activities of blackthorn leaves (232).

*P. spinosa* leaf extracts in water and ethanol were effective against the tested fungal and bacterial strains. *B. cereus* and *E. cloacae* were the most vulnerable bacterial strains, whereas *P. ochrochloron*, *P. funiculosum*, and *T. viride* were some of the most impacted fungal pathogens (232).

According to Veličković et al. (232), blackthorn leaf extracts also showed anti-diabetic activities. The analyzed extracts showed a significant role in blocking the enzymes glucosidase and amylase, which are associated with type II diabetes. In addition, the results shown here are consistent with those of Popović et al. (422) who studied blackthorn fruit extracts.

Furthermore, not much research has been done on the anticancer effects of blackthorn leaf extracts. The ethanol leaf extract had an impact on malignant human cell lines (K562, MDA-MB-453, HeLa), according to the results of Veličković et al. (232).

#### 3.5.3 Peach (*Prunus persica*)

The traditional uses of *Prunus persica* in folk medicine highlight its multifaceted therapeutic potential. *Prunus persica* flowers were historically used to treat rashes and eczema. The leaves served various purposes, functioning as anthelmintic, insecticidal, laxative, sedative, and vermicidal agents, and were also used to address conditions like piles, leucoderma, and whooping cough. The fruits were utilized for their properties as aperients, aphrodisiacs, antipyretics, demulcents, antiscorbutics, and brain tonics. Additionally, the oil extracted from the seeds was considered abortifacient and used to treat ailments such as piles, deafness, earache, and stomach troubles in children (300).

The leaves of *Prunus persica* contain a diverse range of bioactive compounds, including caffeic acid derivatives, p-coumaric acid derivatives, chlorogenic acid, rutin, kaempferol-3-O-rutinoside, kaempferol-3-O-glucoside, kaempferol-3-O-rhamnoside, caffeic acid, quercetin, kaempferol, quercetin-3-glycoside, quercetin-3-rhamnoside, quinic acid, tannins, urosolic acid, and zeaxanthin. These compounds contribute to the medicinal properties and antioxidant activity of *Prunus persica* leaves, making them valuable for various health-related applications (305, 423). In terms of gastrointestinal impact, the aqueous crude leaf extract stimulates gut activity, mediated by cholinergic mechanisms and calcium channel blockade (233). Anti-inflammatory, analgesic, and antipyretic effects are observed, with the methanol extract inhibiting inflammatory mediators and cytokines (234). Cytotoxic effects against tumor cells are noted, attributed to compounds isolated from the leaves (235, 236). Antioxidant properties are evident, with the leaves showing scavenging activity against free radicals (235, 424, 425). Antiparasitic activity is demonstrated against various parasites (237, 238). Anti-obesity effects are seen through the modulation of lipid metabolism (239–241). Anticholinesterase effects offer potential in dementia treatment (242, 243). Furthermore, the leaves exhibit antidiabetic effects through glucose absorption inhibition (244–246). Additionally, dermatological effects include protection against UV-induced damage (247–249).

#### 3.5.4 Olive tree (*Olea europaea* L.)

*Olea europaea* L., a prominent fruit tree species in Mediterranean regions, covers 8 million hectares and dominates 98% of global cultivation, with olive oil, especially extra virgin olive oil (EVOO), being renowned for its health benefits attributed to polyphenols (254). However, olive leaf extract (OLE) surpasses EVOO in polyphenol diversity and quantity, thanks to its distinct structural properties, offering unique potential for promoting health. Researchers are increasingly exploring OLE’s advantages due to its diverse polyphenols, making it a subject of ongoing health-enhancing research (254). A study by Benavente-Garcia et al. identified primary phenolic compounds in olive leaf extracts, demonstrating their antioxidant activities (426). Olive phenols displayed synergistic radical-scavenging capacity in olive-leaf extracts. Flavonoids with more hydroxyl groups were particularly effective in quenching radical cations. Olive leaves contain valuable secoiridoid compounds like oleuropein and oleacein, which are key contributors to their health benefits, particularly in lowering blood pressure. Oleuropein and its metabolite, hydroxytyrosol, possessed optimal antioxidant properties due to their catechol group. They could neutralize various radicals, including hydroxyl, DPPH, and others (427). Oleuropein is also renowned for its antimicrobial, and anti-inflammatory properties, among others (250). In addition to oleuropein, olive leaf extracts contain triterpenes, sterols, erythrodiol, uvaol, and oleanolic acid, with the content varying among different olive leaf types. Oleanolic acid and its isomer, ursolic acid, are significant components, with potential antioxidant benefits (427). Other groups of phenolic compounds found in olive leaves include flavones, flavonols, flavan-3-ols, and substituted phenols (250).

Phenolic compounds derived from olive leaves, including caffeic acid, verbascoside, oleuropein, luteolin 7-O-glucoside, rutin, apigenin 7-O-glucoside, luteolin 4’-O-glucoside, and hydroxytyrosol, have demonstrated the ability to inhibit the growth of various bacteria such as *Escherichia coli*, *Klebsiella pneumoniae*, and *Staphylococcus aureus* (251). Pereira et al. (94) used aqueous extracts of olive leaves to investigate the antimicrobial activity against the Gram− bacteria *Klebsiella pneumoniae*, *Pseudomonas aeruginosa*, and *Escherichia coli*; the Gram+ bacteria *Staphylococcus aureus*, *Bacillus subtilis*, and *Bacillus cereus*; and the fungi *Cryptococcus neoformans* and *Candida albicans*. According to the microbial growth rates in the presence of various concentrations of the extract, olive leaves are arranged in increasing order of antimicrobial capacity: *Bacillus subtilis* < *Pseudomonas aeruginosa* ~ *Klebsiella pneumoniae* ~ *Cryptococcus neoformans* < *Staphylococcus aureus* < *Escherichia coli* < *Candida albicans* ~ *Bacillus cereus*. *Candida albicans* (fungus) and *Bacillus cereus* (Gram+) were the most susceptible organisms to leaf extracts. Indeed, the chemical composition of the olive leaf extract modulates the demonstrated antimicrobial effects. The high content of oleuropein and other phenolic compounds in the extract contributes to its antimicrobial properties (94). Oleuropein and hydroxytyrosol inhibit or slow the growth rate of several human respiratory or enteric tract pathogens, namely *Vibrio arginolyticus* and *V. cholerae*, *Staphylococcus aureus*, *Vibrio parahaemolyticus*, *Salmonella typhi*, *Moraxella catarrhalis*, and *Haemophilus influenzae* (252). Olive leaf may be useful where prolonged use of antibiotics promotes opportunistic infections and is particularly effective against *Pseudomonas* and *Klebsiella*, two genera of bacteria that cause severe resistance problems (253).

Olive polyphenols are considered to play a substantial protective role in cancer and other inflammation-related diseases. Research involving both inflammatory and cancer cell models has revealed that polyphenols derived from olive leaves possess anti-inflammatory properties and the ability to shield against DNA damage caused by free radicals. These multifaceted bioactive properties of olive leaf polyphenols provide a plausible explanation for their capacity to impede the progression and development of cancers. The mechanisms by which olive leaf polyphenols exert their effects include the modulation of the NF-κB inflammatory response and the oxidative stress response. Additionally, their structural similarity to estrogen suggests a potential interaction with estrogen receptors, which could contribute to reducing the incidence and advancement of hormone-related cancers. It is important to note that although there is anecdotal evidence supporting the protective effects of olive polyphenols against cancer in humans, clinical trials are necessary to substantiate these claims (254).

In a pioneering study, the potential neuroprotective effects of standardized dry OLE were investigated for the first time. The research employed a model of transient global cerebral ischemia in Mongolian gerbils to evaluate the impact of OLE on various parameters related to oxidative stress and neuronal damage in the hippocampus. OLE effects were compared with those of quercetin, a well-known neuroprotective plant flavonoid given at the same dosage. The results revealed that pretreatment with OLE significantly reduced the production of superoxide and nitric oxide, lowered lipid peroxidation levels, and boosted the activity of superoxide dismutase across all the observed time points. Furthermore, OLE led to histological improvements by reducing neuronal damage specifically in the CA1 region of the hippocampus. Notably, the neuroprotective effects of OLE were notably stronger than those of quercetin (255).

Systematic reviews, meta-analyses, and research studies were conducted to evaluate the effects of olive leaf extracts supplementation on various cardiovascular-related factors, and are attributed to the primary components of olive leaves, namely oleuropein and oleacein. OLE supplementation was linked to a significant reduction in triglyceride levels and was associated with a decrease in systolic blood pressure (SBP). Subgroup analyses further demonstrated notable improvements in both SBP and diastolic blood pressure in individuals with hypertension (256). In a study by Somova et al. (257), the effects of oleanolic acid, ursolic acid, and extracts from olive leaves sourced from various origins were explored using a genetic rat model of hypertension. Insulin-resistant rats exhibited elevated blood glucose levels and were predisposed to early atherosclerosis, characterized by substantial rises in total cholesterol, LDL cholesterol, and triglycerides. After treatment with oleanolic acid, ursolic acid, or OLE, most of these biochemical parameters were nearly normalized. All samples displayed potent hypoglycemic, antihyperlipidemic (antiatherosclerotic), and antioxidant activities. Studies conducted in alloxan-diabetic rabbits treated with oleuropein demonstrated a notable decrease in blood glucose levels, suggesting that oleuropein may help inhibit hyperglycemia and reduce oxidative stress associated with diabetes. This potential effect could contribute to the prevention of diabetic complications (258, 259). Additional research explored the impact of olive leaf extract on post-meal blood glucose levels in diabetic rats, revealing that compounds like luteolin and oleanolic acid within the extract had inhibitory effects on postprandial blood glucose spikes (260). Moreover, a study involving human subjects showed that oleuropein facilitated the uptake of glucose by cells (261). Notably, oleuropein also acts as an agonist for TGR5, a G-protein-coupled receptor activated by bile acids. TGR5 plays a role in various cellular and physiological processes, and its activation by oleuropein and related compounds may contribute to their antihyperglycemic effects. Bile acids, which activate TGR5, are emerging as important metabolic signaling molecules with the potential to improve metabolic disorders such as obesity and insulin resistance (259). Doxorubicin (DXR), an anthracycline antibiotic widely used in cancer treatment, is highly effective against various malignancies. However, its clinical application is often limited by severe cardiotoxic side effects that can lead to congestive heart failure. The study conducted by Andreadou et al. (262) revealed that all groups treated with oleuropein exhibited minimal cytoplasmic vacuolization in cardiomyocytes compared to the group treated with DXR alone. This suggests that oleuropein protects against DXR-induced cardiotoxicity. Oleuropein achieves this protective effect by effectively inhibiting the production of lipid peroxidation products, reducing oxidative stress, and decreasing the levels of nitric oxide species in cardiomyocytes.

## 4 Possible applications of leaves in the food industry

In recent years, the production of superfoods, functional food, food supplements, nutraceuticals, and body care products has notably increased due to the growing demand from consumers. Having established that fruit and vegetable waste materials are an important source of various bioactive compounds, they have begun to be used to produce new and innovative products in various industrial sectors. For example, berries are a large group of functional foods or “superfoods” offering numerous health benefits, but little is known about their wastes such as leaves. Berry leaves have been widely used by folk medicine to make teas and infusions that have shown beneficial properties for human health, as described in the study by Ferlemi and Lamari (270). The traditional uses of berry leaves are well documented, as already reported in Table 2, but these traditional remedies have been almost completely forgotten. In recent years, the European Medicines Agency (EMA) has approved infusions and extracts of the leaves of *Arctostaphylos uva-ursi*, *Rubus idaea,* and *Ribes nigrum* for distribution as herbal medicines based on traditional use (317–320). Analytical studies have shown that the phenolic composition of leaves is similar or superior to that of valuable fruits, making it useful as an alternative source of phytochemicals for the development of cosmetic products, nutraceuticals, food supplements, or functional foods (270). The interest in the reuse of fruit and vegetable waste is growing a lot in the food industry since the great potential of this waste is known. The use of fruit tree leaves as a source of antioxidants was given very little attention so far. This may be due to their low popularity and lack of commercial applications. Fruit tree leaves have been used in folk medicine to treat various skin conditions (276) and for their antidiarrheal, antipyretic, sedative, and other properties listed in Table 2.

The market for flavorings, fragrances, and flavors has increased due to the growing consumer demand for natural, familiar, and safe sources. In this sector, fruit and vegetable wastes can serve as a source of aromas and flavors in the production of many foodstuffs (28). Strategies are constantly being sought to increase the quality and shelf life of food products, often by limiting or inhibiting oxidative damage. The addition of antioxidants is one of the strategies to slow down the oxidation of biomolecules. Natural antioxidants readily available from natural sources such as fruit and vegetable waste (leaves, seeds, pulp, etc.) have great potential to be used as preservatives in place of their synthetic counterparts (428). The choice of antioxidants for inclusion in foods is subject to specific national regulatory laws or international standards. Synthetic antioxidants are used instead of their natural counterparts because they are more stable, cost-effective, and widely available. Tert-butylhydroquinone (TBHQ), propyl gallate (PG), butylated hydroxytoluene (BHT), and butylated hydroxyanisole (BHA) are the most commonly used synthetic antioxidants in the food industry. In addition, 2,4-dichlorophenoxyacetic acid (2,4-DA), 4-phenylphenol (OPP), and 2-naphthol (2NL) are commonly used in fruits and vegetables. Several studies published in the literature have shown an association between the long-term assumption of synthetic antioxidants and several health problems such as an increased risk of cancer, gastrointestinal problems, and skin allergies (429–431). The trend to replace these antioxidants with natural ones is increasing. In addition, the use of natural antioxidants allows manufacturers to meet the demand for products with unique natural ingredients. Beyond being the main plant compounds with antioxidant activity, some phenolic compounds also have antibacterial and antifungal activity and have an important effect on the taste and texture of foods. Many compounds can inhibit the oxidation of foods, but only some of them are suitable for human consumption for safety reasons. Antioxidants for food use must be approved by regulatory bodies [generally recognized as safe (GRAS)] (432). Natural antioxidants, deriving from leaves and other waste of agricultural production, are a valid alternative to synthetic ones, obviously used within regulatory limits. In recent years, researchers have been investing in this aspect to produce functional foods with added value. To use natural antioxidants in food, they must be approved by the US Food and Drug Administration (FDA) or the European Food Safety Authority (EFSA). Natural antioxidants derived from agricultural wastes have been applied to meat products and to edible oils to improve their stability. They have also been studied in baked goods, and most of the published studies on the use of natural antioxidants show excellent results in the inhibition and control of oxidative processes (432). Research by Michel et al. (433) revealed a link between the phytochemical composition and antioxidant activity of Seabuckthorn (SBT) extracts. The antibacterial activity of three components of SBT, including leaves, seeds, and pomace, was examined by Arora et al. (434). They discovered that the extract from leaves was the most efficient, inhibiting 16 of the 17 reference strains tested. Utilizing SBT for the targeted activation of probiotic bacteria in dairy products is one of the most recent advances. In yogurt samples with 2% of SBT, counts of *Lactobacillus acidophilus* and *Bifidobacterium lactis* were considerably higher, according to research by Gunenc et al. (435). A similar finding was found with the starting culture’s *Lactobacillus bulgaricus* and *Streptococcus thermophilus* components (436). In a study conducted by Salta et al. (437), they enriched sunflower, palm, and olive oils with olive leaf extract containing 200 mg/kg polyphenols. The primary objective of these researchers was to evaluate the oils’ antioxidant capacity and resistance to oxidation in comparison to oils enriched with commercial additives. They employed two methods for this assessment: the 1,1-diphenyl-2-picrylhydrazyl (DPPH) radical scavenging activity test and the Rancimat method to gauge oxidative stability. The findings revealed a significant enhancement in both the antioxidant capacity and oxidative stability of the oils following enrichment with olive leaf extract. This improvement was attributed to the presence of phenolic compounds such as oleuropein, hydroxytyrosol, and quercetin in the extract. Furthermore, the study noted that the olive leaf extract exhibited antimicrobial properties in food products, contributing positively to their overall quality and shelf-life. In another investigation by Gök and Bor (438), they examined the impact of methanolic olive leaf extract at concentrations of 500 and 1,000 ppm on the quality of meatballs during a ten-day storage period. Normally, raw meatballs stored at 4°C have a shelf-life of approximately seven days, with microbial spoilage being a major concern. In control samples, the total aerobic bacteria count exceeded the acceptable limit after ten days of storage. Conversely, meatballs treated with olive leaf extract at concentrations of 500 and 1,000 ppm exhibited significantly lower bacterial counts. Moreover, various other microbial counts, including psychrophilic, *Pseudomonas*, lactic acid bacteria, *Enterobacteriaceae*, yeasts, and molds, were also lower in meatballs containing olive leaf extract compared to the control group. Consequently, the study suggested that olive leaf extract can effectively function as a natural antioxidant and antimicrobial agent, leading to improved overall quality and extended shelf-life of meat products like meatballs (161).

Pitanga (*Eugenia uniflora* L.) leaf extracts applied at dosages of 250 mg/kg delayed the discoloration of the burgers and slowed down protein and lipid oxidation during storage in raw lamb burgers (439). It is important to draw attention to the findings of pitanga extracts, which had the lowest TBAR and carbonyl levels while retaining the sensory properties. Volatile chemicals were also used to assess changes in the secondary byproducts of lipid oxidation. In samples treated with these natural extracts, there were decreased concentrations of hexanal, the most reliable biomarker of lipid oxidation. Therefore, by substituting natural antioxidants for synthetic ones, these extracts provide a viable option for extending the shelf life of meat products (440).

Antioxidant capabilities of guava leaf extracts have been investigated as food preservatives to replace synthetic antioxidants. The guava leaf extract effectively prevents oxidation in fresh pork sausage at 4,000 ppm or higher (441). Antioxidants derived from guava leaves were recommended for further integration into pork sausage in the study by Tran et al. (442). The inclusion of antioxidants at 4,000 ppm delays lipid oxidation and preserves the proper color attributes, being equal to the synthetic antioxidants typically used. This was done to avoid the addition of synthetic antioxidants, and by doing so, the potential health risks associated with the use of synthetic antioxidants should be reduced. Additionally, it is crucial to remember that this research entailed the inclusion of this antioxidant inside a complicated matrix and the significance of preventing any changes to the product’s quality and organoleptic criteria (443). Adding apple leaves to a cloudy apple drink can increase polyphenol content concentration compared to cloudy apple juice. In the study conducted by Kolniak-Ostek et al. (444), the effect of adding apple leaves on viscosity, turbidity, turbidity stability, the composition of phenolic compounds, antioxidant activity, and color properties was examined. These parameters were analyzed to evaluate the potential application of new preparation methods in the production of cloudy apple drinks. The addition of apple leaves was shown to increase viscosity and turbidity in all samples tested. In addition, 15 compounds from four different phenolic groups were found: dihydrochalcones, hydroxycinnamates, flavonols, and flavan-3-ols. The total content of phenolic compounds in the control sample was significantly lower than in the leaf-containing drink. Therefore, the addition of leaves increased the content of polyphenolic compounds. This study suggests that the use of apple tree leaves may be a better and cheaper source of bioactive compounds and may have implications for the prevention of free radical-related diseases. In addition, leaves have important biological and pharmacological properties (444). Based on this evidence, the leaves of the fruits could continue to be used to produce herbal teas or infusions to be consumed daily.

As an alternative to being incorporated in food formulations as natural antioxidants, leaves are used to create protective barriers that are applied directly to food surfaces (edible coatings) or films for food packaging. These barriers represent a new approach to addressing the detrimental effects of oxygen on food (445) and act as antimicrobial barriers. These coatings can extend the shelf life of food products. Extracts from agricultural by-products have shown great potential for inclusion in packaging materials with promising results. Despite being rich in phenolic compounds, glucosides, and gallic acid, mango leaf extract has not received much attention (446). The Rambabu et al. (447) research assessed the addition of mango leaf extract (1, 3, and 5%) to chitosan and the subsequent use of the active system on cashews. The overall phenolic content of the films rose as the extract level rose, as was to be predicted. The oxidation of cashews was more effectively controlled by 3 and 5% enriched films, according to peroxide values tracked for 28 days. Also Baranauskaite et al. (448) prepared a chitosan film containing mango ethanolic leaf extract (MLE) with antioxidant properties. When used as a packaging material for the storage of cashew nuts, fatty acid oxidation was reduced by 56% in nuts stored with 5% MLE films compared to commercial non-biodegradable polyamide/polyethylene (PA/PE) films (432). Moudache et al. (449) study concentrated on olive leaves and extracts from cakes that are high in phenolic compounds such as oleuropein, luteolin, and hydroxytyrosol. Following the selection of the best extraction technique, the most intriguing antioxidant extract (ethanol: water: 70% olive leaf extract) was diluted in an aqueous adhesive solution and used to create two different types of multilayer films: PE-based films (PE/PE) (1 and 2% of extract) and PE/paper films (PE/P) (1 and 2% of extract). The scavenger activity of the active films was assessed by subjecting the films to a gas high in free radicals; PE/PE (10% olive leaf extract) had the greatest performance in terms of antioxidant activity.

Recently, olive pomace and leaf extracts have also been used to enhance the coating films’ characteristics. Khalifa et al. (450) created active chitosan coatings using leaf or pomace extract (1 and 2%) and then compared them to plain chitosan coatings and aqueous wax coatings with thiabendazole. Strawberries, a fruit chosen for its quick kinetic decomposition, were coated with these coatings using a sprayer. The total phenolic content of uncoated goods declined quickly throughout the 16 days of chilled storage, compared to fruits with active coating (the highest impact with 2% of leaves extract). These findings were also supported by a later investigation by the same authors (451), who coated apples with a similar active substance and tracked their efficacy for 35 days while they were kept at 4°C. Additionally, the greatest results were obtained with 2% of the olive leaf extract in this example, which greatly decreased the loss of total phenols and flavonoids in the apples. Licciardello et al. (452) investigated the release kinetics of extracts from active packaging films in three distinct food imitating systems: water, 10% ethanol, and 50% ethanol, in addition to examining the antioxidant activity of by-product extracts. Olive leaf extract and grape pomace extract, two active chemicals, were coated on shellac and cellulose nitrate films for this purpose. The study underlined that release is heavily reliant on the solvent in contact with the film and noted the two extracts’ antioxidant capability. Kiwi leaf extract’s antioxidant strength was also established. The study found that it can shield cells from free radicals caused by physicochemical agents. So, according to Cyboran et al. (355), this extract may be a possible food additive that prevents lipid oxidation in foods that have been exposed to ultraviolet B (UVB) radiation.

Paper is a biodegradable material with average mechanical qualities, but its usage is restricted by its large porosity and poor barrier capabilities against gas and moisture. Kasaai and Moosavi (453) isolated and employed hydrophobic substances from mandarin peel and leaves to treat food-grade Kraft paper to enhance the water vapor and gas characteristics of paper. The authors claimed that some extract fractions filled the paper’s pore spaces while other fractions formed a thin coating on the surface. As a result, compared to the control system, the treated paper’s water vapor permeability, air transmission rate, and peroxide value were all dramatically reduced due to the extracts’ hydrophobic substance, which was later determined to be terpene hydrocarbons. Citrus by-products enhanced both the barrier characteristics and the stability against oxidation, as evidenced by the fact that even the antioxidant activity rose as the extract concentration increased (454).

### 4.1 Possible application of leaves in the pharmaceutical and cosmetic industry

**Figure 3:**
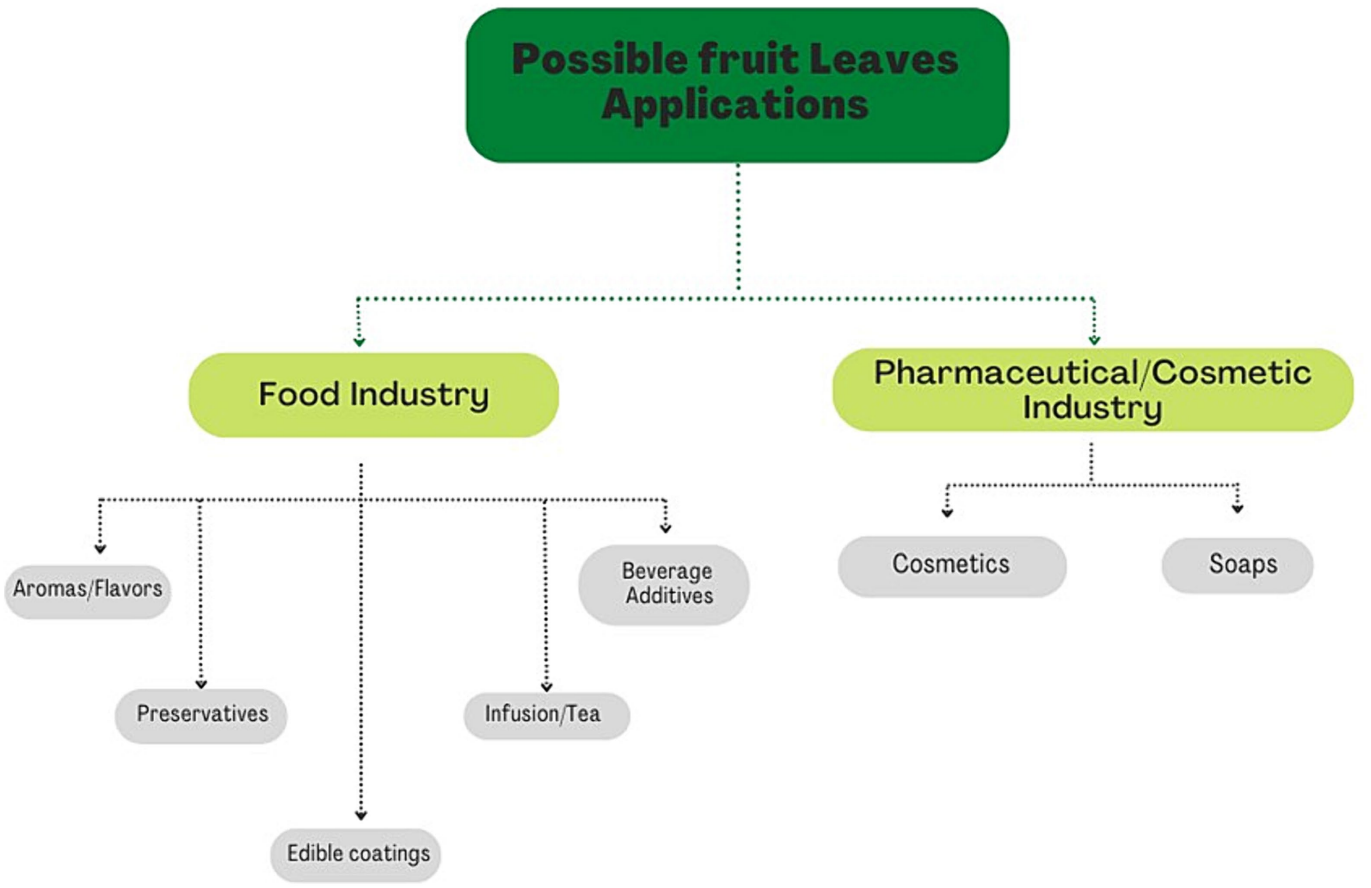
Summary scheme of possible applications of leaves.

Currently, the concept of CE has also been extended to the pharmaceutical and cosmetic industries due to a growing interest in using agricultural waste resources to produce valuable compounds. The leaves of *Fragaria vesca* L., *Rubus fruticosus* L., *Ribes nigrum* L., and *Vaccinum myrtillus* L. belong to the agricultural waste of the berry industry and, from an economic and environmental point of view, many researchers showed that by-products are worth considering as compounds that could be widely used by these industries. The rising expectations of consumers and their desire for cosmetics that are not only beneficial to health but also positively impact the environment are helping to evaluate the potential of using plant waste such as leaves. It has been shown that the extracts of the leaves of the above-mentioned berries have had a positive effect on fibroblasts and keratinocytes; hence, they are promising ingredients in anti-aging cosmetics such as oils, serums, and creams (455). Women frequently utilize the leaves of black mulberry (*M. nigra*) as an alternative to traditional hormone replacement treatment after menopause, producing results comparable to those of estrogen usage (456). Furthermore, due to its extraordinarily strong radical scavenging capacity, its fruits, roots, and leaves extracts may be used in cosmetics all over the world and are a frequently used component in many dermatological creams, bath gels, and other products (457). *Prunus domestica* is the most commonly cultivated plum in Europe, and the chemical composition of its leaves, rich in phenolic compounds, correlates with photoprotective effects and anti-aging and antioxidant activities. Stierlin et al. (458) first reported the promising anti-aging activity of plum leaves due to their ability to inhibit lipoxygenase, hyaluronidase, elastase, and DPPH. Therefore, this study revealed the potential of plum leaf by-products as cosmetics or food supplements. French research confirmed the use of plum leaves in the cosmetic industry (459). For example, Brignoles prune leaves and skin have been included in agricultural by-product screening for cosmetic potential. The anti-aging properties of organic solvent extracts from approximately 30 plants and plant by-products have been investigated. As a result of this study, the crude ethanolic extract of *P. domestica* leaves demonstrated the highest unparalleled anti-aging activity and was subsequently selected for further research, leading to the development of innovative anti-aging actions. Bioactive plant extracts should not normally be incorporated directly into cosmetic formulations, as they may exhibit undesirable properties (viscosity, odor, color, etc.) (459). Wijetunge and Perera (460) used pomegranate (*Punica granatum*) leaf extracts to make soaps with antioxidant and/or antibacterial activity. This study showed that the methanolic Soxhlet extract of pomegranate leaves can be an effective alternative to expensive and seasonal pomegranate fruits for the preparation of beneficial soap products. Liquid medicinal soaps made with this extract retained both the original antioxidant and antibacterial activity shown by the extract, while solid medicinal soaps only exhibited antioxidant activity. In another research, the goal was to create oleuropein-enriched extracts (O20 and O30), a bioactive compound derived from olive byproducts, and add high cosmetic value to them. To do this, a thorough characterization was carried out using high-performance liquid chromatography coupled with mass spectrometry, and their bioactivity was assessed using *in vitro* assays. Forty-nine different chemicals were determined in all, with oleuropein and its derivatives being mostly found in the O30 extract while iridoids were primarily found in the O20 extract. In addition, 10 bioactive compounds were found in olive leaves for the first time. Although O30 displayed greater values, both extracts showed robust antioxidant and antiradical activity. In addition, O30 had higher results for the scavenging of radical oxygen and nitrogen species as well as for the inhibition of enzymes, except for the HOCl and hyaluronidase tests. Regarding cell viability, keratinocyte viability was not decreased by olive byproduct extracts till 100 g/mL. The present study’s findings are all indicative of the potential of industrial wastes as cosmetic components (461). Mango leaf extract (*Mangifera indica* L.) has always been used in cosmetics because of its anti-aging activity and the ability to protect the skin from UV rays and prevent or reduce the effects of heat stress on hair, lips, and skin (462). The high content of mangiferin and other phenolic compounds in the *Mangifera indica* leaf extract indicates its potential use for pharmaceutical, cosmetic, and food purposes (446) (Figure 3). A patient who received a tablet of *C. papaya* leaf extract three times per day for five days saw an increase in platelet count, and it was hypothesized that this effect might be attributed to the expression of the platelet-activating factor receptor gene, which is responsible for the creation of new blood cells (463). Another study has revealed that the leaves of *C. papaya* have a potential impact on boosting a dengue patient’s platelet counts (464). A study found that distinct components of the *C. papaya* plant’s latex, leaves, and seeds have varied beneficial qualities. The proteolytic enzyme papain, which is utilized as a component in the panafil ointment, is found in the latex of the *C. papaya* plant. Due to its ability to cure wounds, this ointment is also used to clean them (465). Carpaine, an alkaloid found in the leaves of *C. papaya*, has amoebic and spasmolytic effects on smooth muscles. According to Mors et al. (466), the papaya plant can be employed in the pharmaceutical business.

## 5 Conclusion

Agriculture is one of the largest producers of by-products, which are defined as secondary products and could have a market value. Examples of FVBs are leaves, kernels, pulp, and seeds, which are sources of numerous high-value-added compounds that can be used as starting materials for the preparation of innovative food products or as natural food ingredients. In recent years, the concept of CE has become important worldwide since it has drastically transformed the “take-make-dispose” paradigm toward a system of recovering and reclaiming the value of natural resources by limiting the overuse of energy and raw materials and avoiding creating useless waste. Thus, in the CE system, what was considered waste becomes a resource, allowing the reuse of important nutrients contained in by-products/food waste. Some of these nutrients are bioactive compounds, produced by plants under stressful conditions, for example, to defend themselves from predators or to survive in the presence of drastic climate changes. These SMs are not directly involved in the vital functions of plants but have a high biological potential that can be exploited in many other fields (food, pharmaceutical, and cosmetic industries). Various studies have shown that leaves have a content of bioactive compounds equal to or greater than fruits. Among SMs, a leading role is played by phenolic compounds, which are known to have beneficial effects on human health, including the prevention of cardiovascular disease and cancer. The leaves have been widely used in folk medicine to treat various ailments such as urinary tract infections, rheumatic pains, or digestive disorders. Over the years, the use of the leaves of fruit plants typical of folk medicine has been lost and various industries have focused on the possibility of obtaining the same effects with synthetic compounds. In recent years, consumers have begun to take an interest in and demand “natural” products leading the industry to reduce the use of synthetic compounds and seek new sources of natural compounds such as leaves. Various bioactive compounds with antioxidant, antimicrobial, or antitumor roles have been isolated from the leaves of fruit plants. These can be used as natural dyes, preservatives, or added to foodstuffs to produce fortified foods. The cosmetic and pharmaceutical industries have also shown interest in these compounds coming from the leaves, using them in the formulation of new cosmetics (creams, serums, soaps), or the production of food supplements. Still today, a lot of information about the leaves from fruit plants remains unknown and, in the literature, there is not yet an appropriate number of studies that focus on understanding the importance and the potential that leaves could have at the industrial level as a by-product of the agricultural industry. Therefore, this review aimed to underline the growing importance of the concept of CE in the industrial field and focused on the beneficial potential that leaves of fruit plants can have, encouraging research to invest in seeking alternative sources of bioactive compounds that are essential for human well-being thanks to their healthy properties. The utilization of waste material as leaves for the exploitation of the beneficial effect of SM can have a double positive effect: firstly, it can allow saving fruits from the “extraction” process for bioactive compounds aimed at cosmetics and pharmaceutics industries, leaving them only for the food and beverage sector; secondly, it can represent an opportunity for the growers to give extra value and re-utilization possibility for an agricultural waste, eliminating or at least reducing some problems related to the agricultural waste management. It is important to understand that, to use these beneficial compounds, it is necessary to develop new organizational systems for the recovery and subsequent reuse of waste materials; without this first step, it will not be possible to take advantage of the beneficial substances deriving from industrial waste. That said, scientific researchers must work hard to create new pieces of evidence for the health benefits of utilizing agricultural waste as a source of bioactive compounds for pharmaceutic and cosmetic industries, considering more fruit plant species, taking into account also agricultural wastes other than leaves (seeds, peel, roots, etc.), and trying to indicate specific end use for different agricultural wastes, working in parallel with the needs of pharmaceutical and cosmetic industries and with consumer expectations.

## Author contributions

LR wrote the initial draft of the manuscript. FG, MB, BM, and LM designed the whole idea of the review article. YA, ME-Z, CM, and KT improved the initial draft of the manuscript. LR and LM undertook the graphical visualization. FG, MB, BM, ME-Z, and LM managed the proofread of the final version of the manuscript. All authors contributed to the article and approved the submitted version.
